# E3 ligase-inactivation rewires CBL interactome to elicit oncogenesis by hijacking RTK–CBL–CIN85 axis

**DOI:** 10.1038/s41388-021-01684-x

**Published:** 2021-02-24

**Authors:** Syed Feroj Ahmed, Lori Buetow, Mads Gabrielsen, Sergio Lilla, Gary J. Sibbet, David Sumpton, Sara Zanivan, Ann Hedley, William Clark, Danny T. Huang

**Affiliations:** 1 Cancer Research UK Beatson Institute, Garscube Estate, Glasgow, UK; 2 Institute of Cancer Sciences, University of Glasgow, Glasgow, UK

**Keywords:** Oncogenes, Ubiquitylation, Ubiquitylation

## Abstract

Casitas B-lineage lymphoma (CBL) is a ubiquitin ligase (E3) that becomes activated upon Tyr371-phosphorylation and targets receptor protein tyrosine kinases for ubiquitin-mediated degradation. Deregulation of CBL and its E3 activity is observed in myeloproliferative neoplasms and other cancers, including breast, colon, and prostate cancer. Here, we explore the oncogenic mechanism of E3-inactive CBL mutants identified in myeloproliferative neoplasms. We show that these mutants bind strongly to CIN85 under normal growth conditions and alter the CBL interactome. Lack of E3 activity deregulates CIN85 endosomal trafficking, leading to an altered transcriptome that amplifies signaling events to promote oncogenesis. Disruption of CBL mutant interactions with EGFR or CIN85 reduces oncogenic transformation. Given the importance of the CBL–CIN85 interaction in breast cancers, we examined the expression levels of CIN85, CBL, and the status of Tyr371-phosphorylated CBL (pCBL) in human breast cancer tissue microarrays. Interestingly, pCBL shows an inverse correlation with both CIN85 and CBL, suggesting that high expression of inactivated CBL could coordinate with CIN85 for breast cancer progression. Inhibition of the CBL–CIN85 interaction with a proline-rich peptide of CBL that binds CIN85 reduced the proliferation of MDA-MB-231 cells. Together, these results provide a rationale for exploring the potential of targeting the EGFR–CBL–CIN85 axis in CBL-inactivated mutant cancers.

## Introduction

Aberrant signaling cascades mediated by receptor protein tyrosine kinases (RTKs) are a characteristic feature of a number of human cancers [1]. The RING ubiquitin ligase (E3) Casitas B-lineage lymphoma (c-CBL or CBL) has a critical role in controlling aberrant and sustained RTK signaling by negatively regulating activated RTKs via their ubiquitination and subsequent proteasomal or lysosomal degradation [2–4].

CBL contains a highly conserved N-terminal tyrosine kinase-binding domain (TKBD) followed by a linker helix region (LHR) and a RING domain [5, 6]. Its E3 activity is tightly regulated by a conserved LHR tyrosine (Tyr371 in CBL). Under normal conditions, this tyrosine is anchored to the TKBD and constrains the RING domain in an inactive conformation. Upon growth factor stimulation, CBL is recruited to an activated RTK, leading to extensive tyrosine phosphorylation in CBL including Tyr371, which induces conformational changes to activate its E3 activity [2–4, 7–10]. In addition, CBL also functions as an adaptor through its C-terminal region. A long stretch of proline-rich residues allows it to interact with SH3 domain-containing proteins such as GRB2, CD2AP/CIN85, Cool/Pix, and others [11–14]. Moreover, upon phosphorylation, C-terminal Tyr700, Tyr731, and Tyr774 act as docking sites for proteins with SH2 domains [15–17]. Thus, under different settings, CBL can have divergent functions as an E3 or an adaptor.

Mutations in *CBL* have been observed in different types of myeloid malignancies accounting for ~5% of such cases [18]. *CBL* mutations are predominantly found in myelodysplastic syndrome/myeloproliferative neoplasms (MDS/MPN), where mutations are clustered within the LHR and RING domains with Tyr371 mutations as the major hotspot [18–21]. These CBL mutants have dominant-negative effect and induce oncogenic phenotypes in various cell lines [22–26]. In addition, conditional knock-in mice expressing CMML-associated CBL-Q367P or ligase-dead CBL-C379A mutant developed CMML-like phenotypes and leukemia, respectively [27, 28]. However, an increasing number of studies suggest that these oncogenic CBL mutants attain gain-of-function properties [22, 27–32], but the exact mechanism remains elusive. It is noteworthy that abnormal CBL expression is associated with the progression of different types of human cancer [33–35], but the status of CBL activation has not been analyzed in these studies.

In this study, we utilized CBL-70Z, a well-characterized oncogenic CBL mutant lacking residues 366–382, and CBL-Y371S, a prevalent MDS/MPN CBL mutant, as E3-inactive CBL mutants to explore their oncogenic mechanism. We found that these mutants predominantly associate with CIN85 (CBL-interacting protein of 85 kDa) to elicit oncogenic transformation. CIN85 is an SH3-containing adaptor protein that associates with CBL and endophilin upon growth factor stimulation to promote trafficking of RTKs to endosomes prior to their lysosomal degradation [2, 3, 36, 37]. Monoubiquitination or phosphorylation of CIN85 is important for this process [38–42]. In contrast to such observations, we show that E3-inactive CBL mutants bind CIN85 in the absence of growth factor stimulation and deregulate CIN85 trafficking and signaling to alter the transcriptome landscape thereby promoting oncogenic transformation. Mechanistically, the cell-transforming ability of these CBL mutants is dependent on CIN85 binding which in turn depends on RTK-mediated phosphorylation of CBL’s C-terminal tyrosines to expose a proline-rich motif for CIN85 binding. Given that CIN85 and CBL have been implicated in the invasiveness of breast cancer cells [43], we examined the expression of CIN85, CBL and Tyr371-phosphorylated CBL (pCBL) in human breast cancer tissue samples. The levels of CIN85 and CBL progressively increase as cancer stages advance. Interestingly, pCBL shows an inverse correlation with both CIN85 and CBL, suggesting that high levels of inactivated CBL could coordinate with CIN85 for cancer progression. Furthermore, we show that a peptide mimicking the proline-rich region of CBL that binds CIN85 reduces the proliferation of breast cancer cells. Our findings provide a rationale for targeting CBL in cancers with CBL mutation or inactivation for therapeutic intervention.

## Results

### Oncogenic CBL mutants interact with CIN85 in the absence of EGF stimulation

**Fig. 1:**
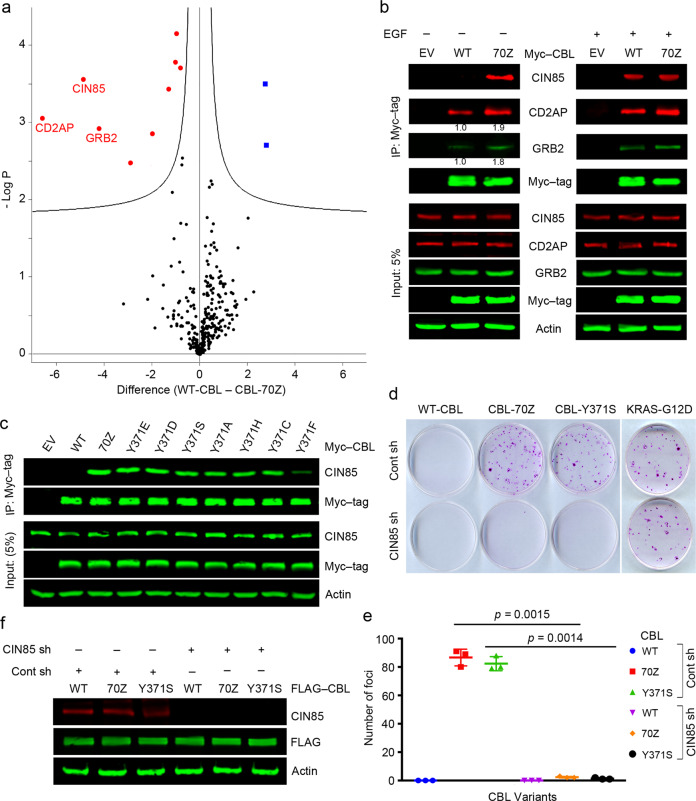
CBL mutants interact strongly with CIN85 to induce cellular transformation. **a** Volcano plot showing differences in interactomes between WT-CBL and CBL-70Z. The experiment was performed in triplicate. Red dots represent significant hits with CBL-70Z as compared to WT-CBL while blue dots represent significant hits with WT-CBL as compared to CBL-70Z. **b** Co-immunoprecipitation assay of HEK293 cell lysates with overexpressed Myc-tagged WT-CBL, CBL-70Z, or empty vector (EV) in the absence (left) and presence (right) of stimulation with EGF. Anti-Myc immunoprecipitates and cell lysates were analyzed by immunoblotting with anti-CIN85, anti-CD2AP, anti-GRB2, anti-Myc tag, and anti-actin antibodies as indicated. In the right panels, the cells were treated with 50 ng/ml EGF for 5 min prior to harvesting. **c** Co-immunoprecipitation assay of HEK293 cell lysates with overexpressed Myc-tagged CBL variants or EV as indicated. Anti-Myc antibody immunoprecipitates and cell lysates were analyzed by immunoblotting with anti-CIN85, anti-Myc tag, and anti-actin antibodies as indicated; *n* = 3 independent experiments. **d** Foci-formation assay using 3T3 cells stably expressing CBL variants or a positive control KRAS-G12D with and without *CIN85* knockdown; 3T3 cells expressing the oncogenic KRAS-G12D were used as a positive control for cell transformation. **e** Quantification of **d**; *n* = 3 independent experiments. Error bars represent ± SEM. **f** Expression of CIN85, FLAG, and actin in 3T3 cells stably expressing FLAG-tagged WT-CBL, CBL-70Z, or CBL-Y371S with and without *CIN85* knockdown from **d**; *n* = 3. The actin panel is a sample control in **b**, **c**, and **f**. Associated with Supplementary Fig. 1 and Supplementary Table 1.

CBL mutants, for example, 70Z, Y371S, and Y371H attain a gain-of-function to drive oncogenesis [22, 30, 31]. We wondered whether the gain-of-function effect of these CBL mutants might arise from different interactomes compared to wild-type (WT) CBL. To test this hypothesis, we overexpressed Myc-tagged WT-CBL or CBL-70Z in HEK293 cells and performed Myc-trap pull-downs followed by mass spectrometry analyses. We found differences between the WT-CBL and CBL-70Z interactomes in the absence of growth factor stimulation (Fig. 1a and Supplementary Fig. 1a, b, Supplementary Table 1). Immunoprecipitation experiments confirmed these observations in the absence of growth factor stimulation. CIN85 displayed a strong interaction with CBL-70Z as compared to WT-CBL while CD2AP and GRB2 to some extent also showed greater interactions with CBL-70Z (Fig. 1b). Upon EGF stimulation, the differences between WT-CBL and CBL-70Z in CIN85, CD2AP, and GRB2 pull-down became less obvious (Fig. 1b). Hereafter, we focused on the effect of CIN85. Next, we assessed whether various CBL-Y371 mutants (Y371S, Y371E, Y371D, Y371A, Y371H, Y371C, and Y371F) displayed similar interactions with CIN85 and found that they all pulled down CIN85 in the absence of EGF stimulation (Fig. 1c and Supplementary Fig. 1c). Likewise, other MDS/MPN CBL mutants such as R420Q and R420L also pulled down CIN85 in the absence of EGF stimulation (Supplementary Fig. 1d). Together, these results showed that in contrast to WT-CBL, CBL mutants acquire the ability to interact with CIN85 in the absence of growth factor stimulation.

### CIN85 interaction is essential for CBL mutant-induced transformation

To assess whether CIN85 could confer oncogenic potential to the CBL mutants, we performed foci-formation assays in the presence and absence of CIN85. Foci formation was observed in cells expressing endogenous CIN85 and stably overexpressing CBL-70Z or CBL-Y371S but not WT-CBL (Fig. 1d–f). Interestingly, both CBL-70Z and CBL-Y371S expressing cells failed to form foci when stably knocked down for CIN85 even though the control oncogenic KRAS-G12D mutant still promoted transformation in the CIN85 knocked down cells. Together, these results indicate that CIN85 is important for CBL mutant-driven transformation.

**Fig. 2:**
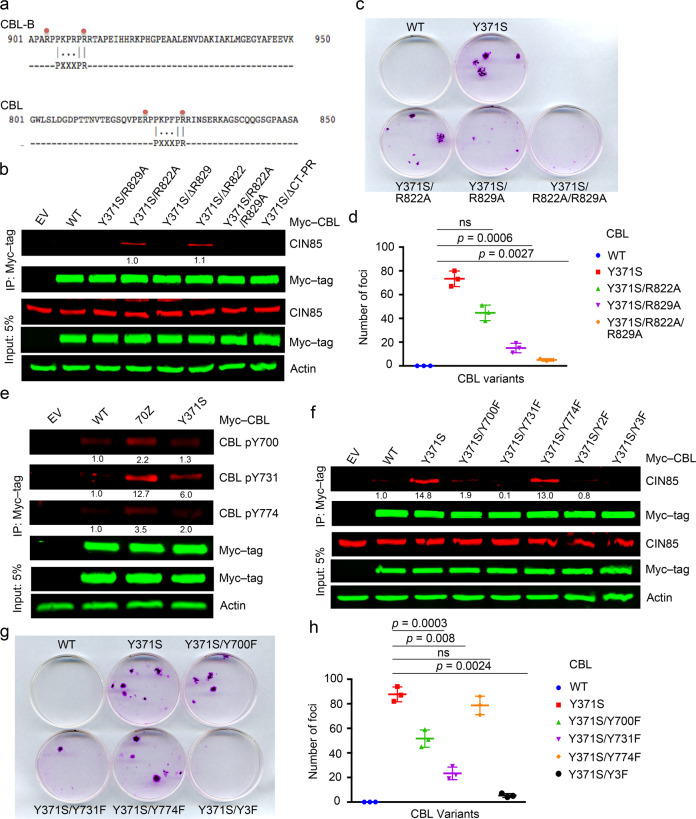
C-terminal tyrosine hyper-phosphorylation of CBL-Y371S is critical for CIN85 binding. **a** Comparison of CIN85-interacting consensus proline-rich motif in CBL-B and CBL. A red dot indicates CIN85-interacting Arg. **b** Co-immunoprecipitation assay of HEK293 cell lysates overexpressing Myc-tagged CBL-Y371S variants or EV. Anti-Myc immunoprecipitates and cell lysates were analyzed by immunoblotting with anti-CIN85, anti-Myc tag, and anti-actin antibodies as indicated. Band intensities of immunoprecipitated CIN85 were quantified and average values from (*n* = 3) independent experiments relative to Y371S/R822A are represented below the bands. **c** Foci-formation assay using 3T3 cells stably expressing WT-CBL or CBL-Y371S variants. **d** Quantification of **c**; *n* = 3 independent experiments. **e** Co-immunoprecipitation assay of HEK293 cell lysates overexpressing Myc-tagged WT-CBL, CBL-70Z, CBL-Y371S, or EV. Anti-Myc immunoprecipitates and cell lysates were analyzed by immunoblotting with anti-CBL pY700, anti-CBL pY731, anti-CBL pY774, anti-Myc tag, and anti-actin antibodies as indicated. Band intensities of immunoprecipitated CBL pY700, CBL pY731, and CBL pY774 were quantified and average values from (*n* = 3) independent experiments relative to WT-CBL are represented below the bands. **f** Co-immunoprecipitation assay of HEK293 cell lysates overexpressing Myc-tagged WT-CBL, CBL-Y371S variants, or EV. Anti-Myc immunoprecipitates and cell lysates were analyzed by immunoblotting with anti-CIN85, anti-Myc tag, and anti-actin antibodies as indicated. Band intensities of immunoprecipitated CIN85 were quantified and average values from (*n* = 3) independent experiments relative to WT-CBL are represented below the bands. **g** Foci-formation assay using 3T3 cells stably expressing WT-CBL or CBL-Y371S variants. **h** Quantification of **g**; *n* = 3 independent experiments. Error bars represent ± SEM and Student’s *t*-test was used to calculate *p*-values in **d** and **h**. The actin panel is a sample control in **b**, **c**, and **f**.

The structure of CIN85’s SH3 domain bound to a proline-rich peptide from CBL-B and mutational analyses show that R904 and R911 of CBL-B and the corresponding R822 and R829 in CBL are important for CIN85 interaction [14]. To verify this CBL–CIN85 interaction, we designed a peptide encompassing the consensus SH3 domain-binding proline-rich PXXXPR motif from CBL (PERPPKPFPRRIN; PepC1) (Fig. 2a). We tested its binding affinity for CIN85 using surface plasmon resonance (SPR) assays and found that PepC1 binds CIN85 full-length with a *K*_d_ of ~5.5 μM (Table 1, Supplementary Fig. 2). We measured the binding affinity of CIN85 SH3 domains: CIN85-SH3A, CIN85-SH3B, and CIN85-SH3C to PepC1 and found that they have *K*_d_’s of ~36, ~2, and 68 μM, respectively, but showed no binding for a control peptide from the C terminus of CBL (IDSCTYEAMYN; PepC2) (Table 1 and Supplementary Fig. 2). Accordingly, we generated secondary site mutations (R822A, R829A, ΔR822, ΔR829, R822A/R829A, and ΔCT-PR lacking residues 822-830) in CBL-Y371S and performed immunoprecipitation experiments. Deletion of R829 or alanine substitution alone was sufficient to disrupt the interaction between CBL-Y371S and CIN85 (Fig. 2b). Correspondingly, cells stably expressing CBL-Y371S with secondary site mutations R829A or R822A/R829A reduced foci-forming ability (Fig. 2c, d). These data further support our finding that interaction with CIN85 is important for cell transformation by CBL-Y371S.

**Table 1:** Dissociation constants (*K*_d_) for interactions between CIN85 FL, CIN85-SH3A, CIN85-SH3B, CIN85-SH3C WT-CBL, CBL-Y371S, PepC1, PepC2, and pY-EGFR peptide.

Immobilized protein	Analyte	*K*_d_ (μM)
CIN85 FL	PepC1	5.5 ± 0.5
CIN85 FL	PepC2	N.M.
CIN85-SH3A	PepC1	35.6 ± 0.7
CIN85-SH3A	PepC2	N.M.
CIN85-SH3B	PepC1	2.3 ± 0.2
CIN85-SH3B	PepC2	N.M.
CIN85-SH3C	PepC1	68.0 ± 15.4
CIN85-SH3C	PepC2	N.M.
WT-CBL (47–435)	pY-EGFR peptide	1.2 ± 0.1
CBL-Y371S (47–435)	pY-EGFR peptide	0.6 ± 0.1

All proteins were immobilized via an N-terminal GST-tag. SEM and dissociation constants (*K*_d_) are indicated. The number of replicates, representative sensorgrams, and binding curves are shown in Supplementary Fig. 2. N.M. indicates no measurable binding.

### C-terminal tyrosine phosphorylation of CBL-Y371S is important for interaction with CIN85

A number of reports showed that oncogenic CBL mutants are tyrosine hyper-phosphorylated [44, 45]. Specifically, Y700, Y731, and Y774 of WT-CBL have been implicated in various signaling processes [15–17] and CIN85 interaction [2, 31]. To assess the phosphorylation status of these three tyrosine residues, we overexpressed Myc-tagged WT-CBL, CBL-70Z, and CBL-Y371S in HEK293 cells and performed immunoprecipitation experiments. All three tyrosine residues particularly Y731 showed enhanced phosphorylation in CBL-70Z and CBL-Y371S compared to WT-CBL (Fig. 2e). To address the relevance of phosphorylation of these tyrosine residues, we generated secondary site mutations (Y700F, Y731F, Y774F, Y700F/Y731F (Y2F), Y700F/Y731F/Y774F (Y3F)) in CBL-Y371S and assessed the ability of these mutants to interact with CIN85. Among the individual mutants, Y731F showed the greatest effect in disrupting the association between CBL-Y371S and CIN85, and incorporation of Y3F resulted in no interaction with CIN85 (Fig. 2f). These data were further corroborated with foci-formation assays in which cells stably expressing CBL-Y371S/Y3F completely lost their ability to form foci (Fig. 2g, h). Thus, the C-terminal tyrosine hyper-phosphorylation of CBL-Y371S is critical for its interaction with CIN85 and cell transformation potential.

### Oncogenic potential of CBL-Y371S depends on its RTK binding

**Fig. 3:**
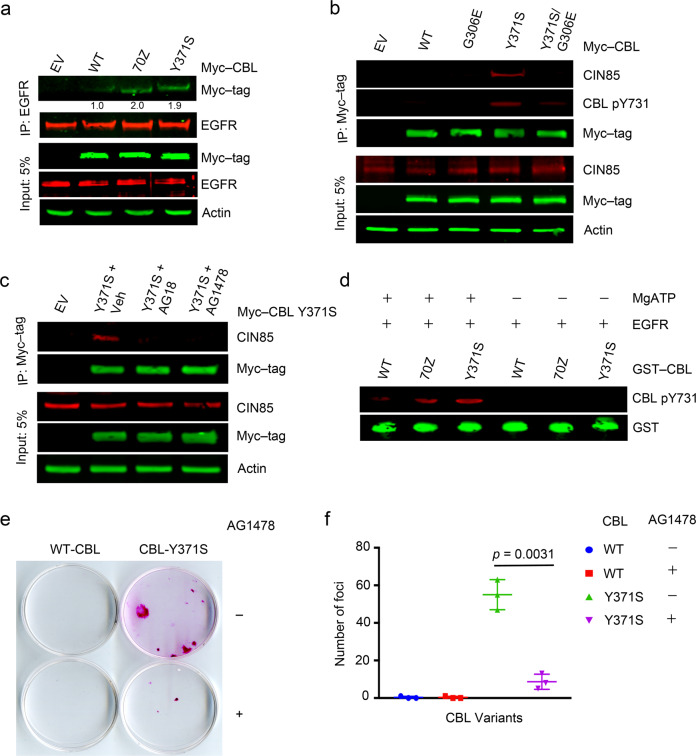
RTK binding is important for the oncogenic potential of CBL-Y371S. **a** Co-immunoprecipitation assay of HEK293 cell lysates with overexpressed Myc-tagged WT-CBL, CBL-70Z, CBL-Y371S, or EV. Anti-EGFR immunoprecipitates and cell lysates were analyzed by immunoblotting with anti-Myc tag, anti-EGFR, and anti-actin antibodies as indicated. Band intensities of immunoprecipitated Myc-CBL variants were quantified and average values from (*n* = 3) independent experiments relative to WT-CBL are represented below the bands. **b** Co-immunoprecipitation assay of HEK293 cell lysates with overexpressed Myc-tagged WT-CBL, CBL-G306E, CBL-Y371S, CBL-Y371S/G306E, or EV. Anti-Myc tag immunoprecipitates and cell lysates were analyzed by immunoblotting with anti-CIN85, anti-CBL pY731, anti-Myc tag, and anti-actin antibodies as indicated; *n* = 3 independent experiments. **c** Co-immunoprecipitation assay of HEK293 cell lysates with overexpressed Myc-tagged CBL-Y371S or EV, treated with vehicle (Veh), 50 μM AG18 or 50 μM AG1478. Anti-Myc immunoprecipitates and cell lysates were analyzed by immunoblotting with anti-CIN85, anti-Myc tag, and anti-actin antibodies as indicated; *n* = 3 independent experiments. The actin panel is a sample control in **a**–**c**. **d** In vitro kinase assay performed with full-length GST-tagged WT-CBL, CBL-70Z, or CBL-Y371S in the presence of purified active EGFR. Phosphorylation was detected by immunoblotting with a CBL pY731 antibody while GST protein levels were used to demonstrate even loading; *n* = 3 independent experiments. **e** Foci-formation assay using 3T3 cells stably expressing WT-CBL or CBL-Y371S with or without AG1478 treatment. **f** Quantification of **e**; *n* = 3 independent experiments. Error bars represent ± SEM. Student’s *t*-test was used to calculate *p*-values. Associated with Supplementary Fig. 3.

Next, we examined how CBL-Y371S becomes hyper-phosphorylated and wondered whether RTK binding was important. We showed that EGFR binds WT-CBL, CBL-70Z, and CBL-Y371S although it was noticeable that more of the CBL mutants were pulled down than WT-CBL (Fig. 3a, Supplementary Fig. 3a). SPR analyses showed that WT-CBL and CBL-Y371S fragments (47–435) bind tyrosine-phosphorylated EGFR peptide (LQRpYSSDPTGA) with *K*_d_’s of 1.2 and 0.6 μM, respectively (Table 1, Supplementary Fig. 2). This enhancement in binding affinity for the CBL-Y371S fragment might explain the differences in amounts of WT-CBL and CBL-Y371S pulled down by EGFR. These results demonstrate that CBL mutants are competent in binding EGFR.

Substitution of G306E in CBL disrupts RTK binding [45], so we incorporated this as a secondary site mutation in CBL-Y371S and investigated its effects on CIN85 interaction. Introduction of G306E into CBL-Y371S reduced Y731 phosphorylation and abolished interactions with CIN85 (Fig. 3b and Supplementary Fig. 3b). Next, we treated HEK293 cells overexpressing Myc-tagged CBL-Y371S with EGFR inhibitors AG18, AG1478, gefitinib, and erlotinib, and performed immunoprecipitation experiments. Treatment with EGFR inhibitors greatly reduced the ability of CBL-Y371S to interact with CIN85 (Fig. 3c and Supplementary Fig. 3c, d). To verify that EGFR can catalyze CBL Y731 phosphorylation, we performed in vitro kinase assays by incubating GST-tagged full-length WT-CBL, CBL-70Z, or CBL-Y371S purified from *E. coli* with purified active EGFR and monitored Y731 phosphorylation. We found that EGFR phosphorylated Y731 of both WT and mutant CBLs in vitro (Fig. 3d and Supplementary Fig. 3e). Furthermore, the foci-forming ability of cells stably expressing CBL-Y371S was significantly reduced when treated with the EGFR inhibitor AG1478 (Fig. 3e, f). Together, these data underscore the importance of RTK binding and RTK-mediated phosphorylation as key steps for CBL-Y371S to become tyrosine hyper-phosphorylated at its C terminus thereby enabling interactions with CIN85 to initiate transformation.

### Oncogenic mutant CBLs are unable to traffic CIN85 to endosomes

**Fig. 4:**
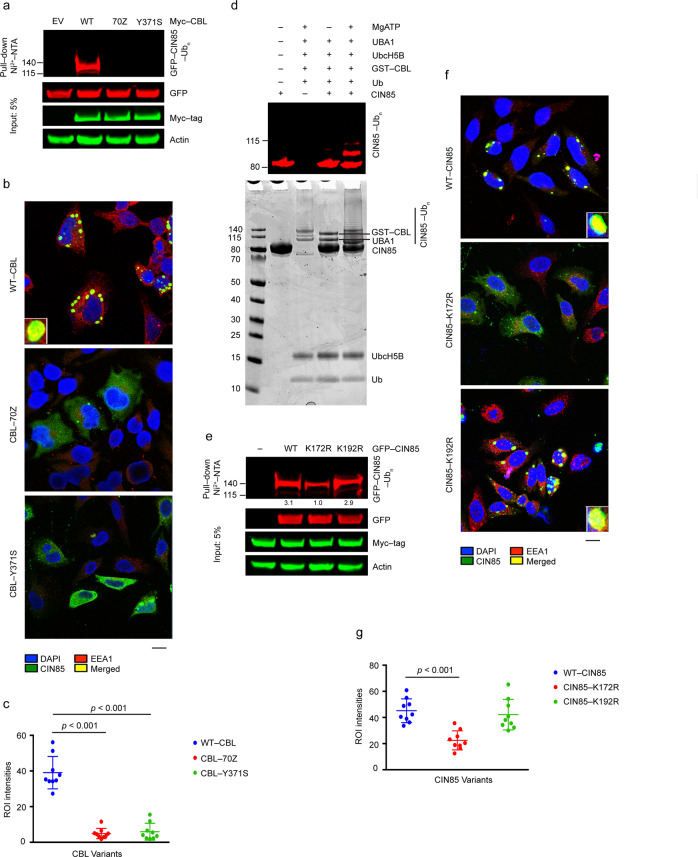
E3 ligase activity-deficient CBL mutants are unable to traffic CIN85 to endosomes. **a** Ubiquitination assay of HEK293 cell lysates with overexpressed Myc-tagged WT-CBL, CBL-70Z, CBL-Y371S, or EV along with GFP-CIN85 and His-Ub. Ni^2+^-pull-down products and the cell lysates were analyzed by immunoblotting against anti-GFP, anti-Myc tag and anti-actin antibodies as indicated; *n* = 3 independent experiments. **b** HeLa cells stably expressing WT-CBL, CBL-70Z, or CBL-Y371S were overexpressed with GFP-tagged CIN85 (green), fixed, and subjected to immunofluorescence staining using antibodies against EEA1 (red) and DAPI (blue, nuclei). Images were captured using confocal microscopy. The box at the left bottom corner shows a zoomed view of co-localized CIN85 and EEA1. Scale bars, 25 μm. **c** Quantification of **b**; *n* = 3 independent experiments. **d** In vitro ubiquitination assay performed with purified full-length CIN85 and GST-tagged CBL. The reaction was run for 30 min at room temperature. The Coomassie-stained gel (lower panel) shows the presence of Ub, UbcH5B, CIN85, UBA1, and GST-CBL. The gel was used for mass spectrometric analyses to identify CIN85 ubiquitinated sites. A fraction of each reaction was used for immunoblotting against anti-CIN85 as represented in the upper panel. **e** Ubiquitination assay of HEK293 cell lysates with overexpressed GFP-tagged WT-CIN85, CIN85-K172R, CIN85-K192R, or EV in the presence of Myc–WT-CBL and His-Ub. Ni^2+^-pull-down products and the cell lysates were analyzed by immunoblotting against anti-GFP, anti-Myc tag, and anti-actin antibodies as indicated. Band intensities of Ni^2+^-pull-down ubiquitinated products were quantified and average values from (*n* = 3) independent experiments relative to CIN85-K172R are represented below the bands. The actin panel is a sample control in **a** and **e**. **f** HeLa cells stably expressing WT-CBL were overexpressed with GFP-tagged WT-CIN85, CIN85-K172R, or CIN85-K192R (all green), fixed and subjected to immunofluorescence staining using antibodies against EEA1 (red) and DAPI (blue, nuclei). Images were captured using confocal microscopy. The box at the right bottom corner shows a zoomed view of co-localized CIN85 variants and EEA1. Scale bars, 25 μm. **g** Quantification of **f**; *n* = 3 independent experiments. The cells in all experiments except **d** were treated with 50 μM chloroquine for 24 h and stimulated with 50 ng/ml EGF for 5 min prior to harvesting or processing for immunocytochemistry. In **c** and **g**, average intensities of the region of interest (ROI) representing the fused early endosomes from three different fields of view for each set have been plotted. Error bars represent ± SEM. Student’s *t*-test was used to calculate *p*-values. Associated with Supplementary Figs. 4–6 and Supplementary Table 2.

Since CBL-70Z and CBL-Y371S lack E3 activity, we investigated how these mutants influence CIN85 ubiquitination and trafficking. To assess the ubiquitination status, lysates from HEK293 cells overexpressing Myc-tagged empty vector, WT-CBL, CBL-70Z, or CBL-Y371S were subjected to cell-based ubiquitination assays. WT-CBL, but neither CBL-70Z nor CBL-Y371S ubiquitinated both exogenous and endogenous CIN85 upon EGF stimulation (Fig. 4a and Supplementary Fig. 4a). In the presence of overexpressed WT-CBL, CIN85 showed a distinct punctate pattern and co-localized with the early endosomal marker EEA1 whereas, in the presence of overexpressed CBL-70Z or CBL-Y371S, it showed a uniform distribution throughout the cell (Fig. 4b, c and Supplementary Fig. 4b). The localization pattern of CIN85 looked similar whether or not cells were treated with the lysosomal inhibitor chloroquine (Supplementary Fig. 4b).

Next, we investigated the lysine residues ubiquitinated by CBL in CIN85. To identify the ubiquitinated lysine sites, we performed an in vitro ubiquitination assay with purified full-length CIN85 and WT-CBL (Fig. 4d) and analyzed the ubiquitinated CIN85 bands using mass spectrometry (Supplementary Table 2). One particular lysine, K172 (Supplementary Fig. 5), gave the highest MaxQuant score. We found that ubiquitination of CIN85-K172R was reduced whereas ubiquitination of CIN85-K192R another site identified through mass spectrometry was similar to WT-CIN85 in the presence of WT-CBL (Fig. 4e). In contrast to WT-CIN85 and CIN85-K192R, CIN85-K172R failed to localize to endosomes efficiently independent of treatment with the lysosomal inhibitor chloroquine (Fig. 4f, g and Supplementary Fig. 4c). However, we find overexpression of CBL causes an increase in the size of the early endosomes (Supplementary Fig. 6) corroborating previous findings of a role for CBL in early endosome fusion [46]. The lack of complete disruption of ubiquitination and endosomal trafficking of CIN85-K172R suggests that there are additional ubiquitination sites. Nonetheless, these data showed that ubiquitination of CIN85 by CBL is important for its endosomal trafficking. Oncogenic CBL mutants lacking E3 activity cannot ubiquitinate CIN85, resulting in a failure of CIN85 trafficking to endosomes.

### Oncogenic CBL mutants activate Cdc42 in a CIN85-dependent manner

**Fig. 5:**
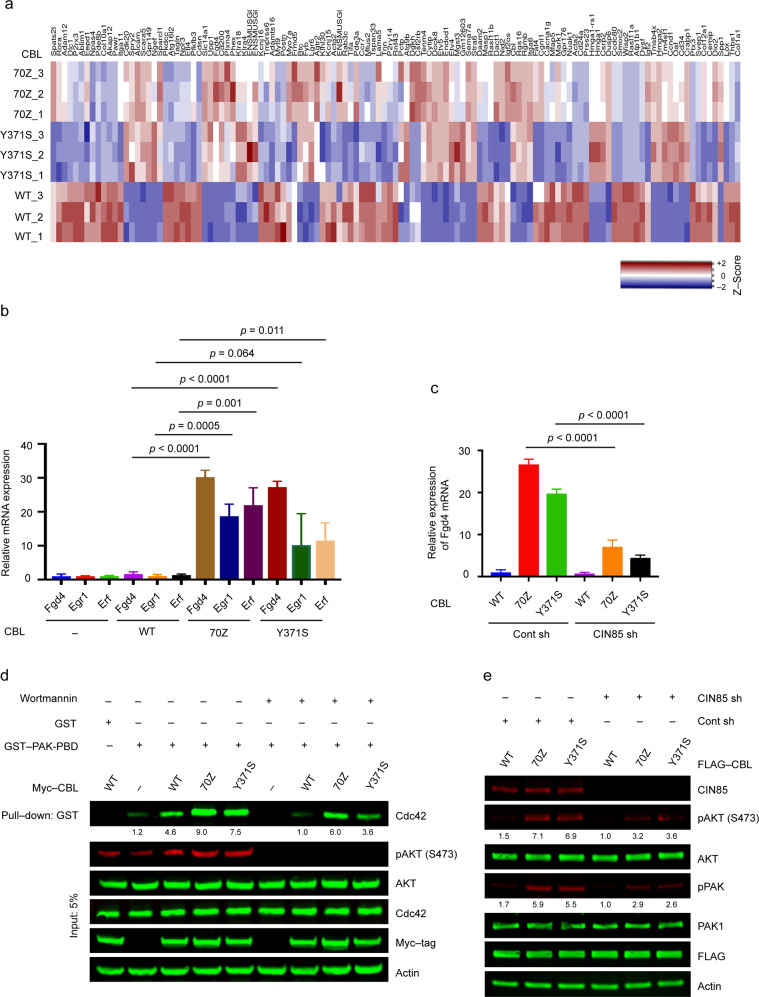
CIN85-interacting CBL mutants activate Cdc42. **a** Heatmap of RNA sequencing data comparing normalized expression of WT-CBL, CBL-70Z, and CBL-Y371S against EV. RNA was isolated from 3T3 cells stably expressing the CBL variants and subjected to RNA sequencing. Red and blue represent up- and downregulated expression, respectively. The experiment was performed in triplicate. **b** Bar graphs showing relative expression of genes from 3T3 cells stably expressing CBL variants. RNA was extracted from 3T3 cells stably expressing WT-CBL, CBL-70Z, or CBL-Y371S and quantitative RT-PCR was performed to measure transcript levels of *Fgd4*, *Egr1*, and *Erf*. The bars represent 95% confidence intervals for relative expression. The experiment was performed in triplicate. Student’s t-test was used to calculate *p*-values. **c** Bar graphs showing relative expression of *Fgd4* with and without *CIN85* knockdown. RNA was extracted from 3T3 cells stably expressing WT-CBL, CBL-70Z, or CBL-Y371S with and without *CIN85* knockdown and quantitative RT-PCR was performed to check *Fgd4* levels. The bars represent 95% confidence intervals for relative expression. The experiment was performed in triplicate. Student’s *t*-test was used to calculate *p*-values. **d** Cdc42 activation assay of HEK293 cell lysates overexpressed with CBL variants in the absence and presence of 1 μM Wortmannin treatment for 24 h. Wortmannin untreated or treated lysates from HEK293 cells overexpressing EV, WT-CBL, CBL-70Z, or CBL-Y371S were incubated with GST-PAK1-PBD bound to glutathione agarose. Immunoblotting was done against active Cdc42 using an anti-Cdc42 antibody. Lysates were also checked for expression of pAKT (S473), pan AKT, CBL variants (Myc tag), and actin. Band intensities of GST-PAK1-PBD pulled down Cdc42 were quantified and average values from (*n* = 3) independent experiments relative to Wortmannin treated WT-CBL are represented below the bands. **e** Expression of CIN85, pAKT (S473), pan AKT, pPAK, PAK1, FLAG, and actin in 3T3 cells from **c**; stably expressing FLAG-tagged WT-CBL, CBL-70Z, or CBL-Y371S with and without *CIN85* knockdown. Band intensities of pAKT (S473) and pPAK were quantified and average values from (*n* = 3) independent experiments relative to WT-CBL/CIN85 sh are represented below the bands. The actin panel is a sample control in **d** and **e**. Associated with Supplementary Table 3.

Next, we asked what signaling events are triggered by CBL mutant-CIN85 complexes to initiate oncogenesis. We examined the transcripts of 3T3 cells stably expressing WT-CBL, CBL-70Z, or CBL-Y371S and observed an inverse relationship in the transcript profiles of cells overexpressing WT and CBL mutants (Fig. 5a and Supplementary Table 3). Among the genes that were significantly upregulated in the presence of CBL mutants was *Fgd4*, a Cdc42 GEF (guanine nucleotide exchange factor). Quantitative real-time PCR confirmed that *Fgd4* transcript levels were upregulated in the presence of CBL mutants as compared to WT-CBL (Fig. 5b). Since FGD4 is a Cdc42-associated GEF, we checked the transcript levels of two Cdc42-responsive genes, *Egr-1* and *Erf*, and found both to be upregulated in the presence of CBL mutants (Fig. 5b). Correspondingly, knockdown of CIN85 led to reduced *Fgd4* transcript levels in cells expressing CBL mutants (Fig. 5c), suggesting that CBL mutant-mediated transcriptional regulation of *Fgd4* was CIN85-dependent. To verify that upregulated *Fgd4* increased Cdc42 activation, we performed pull-downs of Cdc42 in the presence of WT-CBL, CBL-70Z, or CBL-Y371S using PAK1 p21-binding domain (PBD), which binds the active GTP-bound state of Cdc42. More Cdc42 was pulled down in the presence of CBL mutants as compared to WT-CBL (Fig. 5d), consistent with higher Cdc42 activation. CBL was shown to interact with the p85 subunit of PI3K through its phosphorylated Y731 [47]. The addition of PI3K inhibitor Wortmannin reduced the levels of activated Cdc42 in cells expressing all CBL variants or empty vector (Fig. 5d). However, cells expressing CBL mutants still exhibited strong Cdc42 activation in the presence of Wortmannin as compared to WT-CBL expressing cells (Fig. 5d). These results suggest that in addition to PI3K, CBL mutants also use a PI3K-independent pathway to activate Cdc42. Consistent with higher Cdc42 activation, we found that phosphorylation of AKT and PAK was elevated in the presence of CBL mutants (Fig. 5e). In contrast, when CIN85 was knocked down, CBL mutant-mediated activation of both AKT and PAK was greatly reduced (Fig. 5e). Together, these results signify the importance of interactions between CIN85 and oncogenic CBL mutants in activating downstream signaling pathways.

### CIN85 shows an inverse correlation with activated CBL in breast cancer

**Fig. 6:**
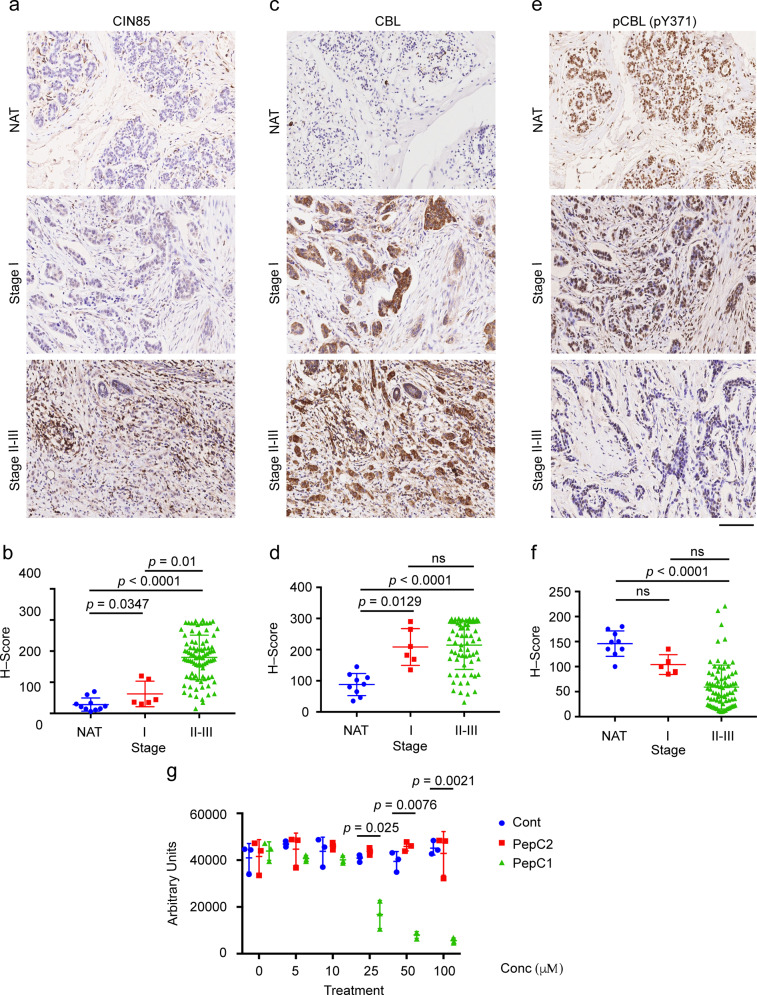
CIN85 shows an inverse correlation with pCBL. **a** Representative examples of CIN85 staining in NAT (*n* = 10), Stage I (*n* = 6), Stages II–III cores (*n* = 91). **b** Quantification of CIN85 expression as shown by CIN85 histoscore (*H*-Score) across different stages for the full tissue microarray (TMA). **c** Representative examples of CBL staining in NAT (*n* = 9), Stage I (*n* = 6), Stages II–III (*n* = 67) cores. **d** Quantification of CBL expression as shown by CBL histoscore (*H*-Score) across different stages for the full TMA. **e** Representative examples of pCBL (pY371) staining in NAT (*n* = 9), Stage I (*n* = 5), Stages II–III cores (*n* = 80). **f** Quantification of pCBL (pY371) expression as shown by pCBL (pY371) histoscore (*H*-Score) across different stages for the full TMA. Only intact cores following immunohistochemistry were used for analyses. Images were exported using Halo software at ×20 original magnification. The scale bar (bottom right) represents 100 µm in each panel. **g** Plot for MDA-MB-231 cell proliferation assay (*n* = 3) using CyQUANT direct showing growth inhibitory properties of PepC1. Cells were treated with 1 μM cell-penetrating Antennapedia peptide in the absence and presence of PepC1 and PepC2 peptides at concentrations of 5, 10, 25, 50, and 100 μM. Student’s *t*-test was used to calculate *p*-values in **b**, **d**, **f**, and **g**. Associated with Supplementary Fig. 7 and Supplementary Table 4.

Given the importance of CBL and CIN85 in cancer progression of breast cancer cells and tissues [34, 43, 48, 49], we investigated their association in breast cancer tissue microarrays (Supplementary Table 4) through immunohistochemistry. The expression of CIN85 was very high between stages II and III as compared to the cancer adjacent normal breast tissue (NAT) and stage I breast cancer tissues (Fig. 6a, b). Similarly, low levels of CBL expression were observed in the NAT but increased in stage I and became higher in stages II–III (Fig. 6c, d). Thus, in advanced stages of breast cancer, CIN85 and CBL could coordinate to promote cancer progression and metastasis. We also looked at the CBL E3 activation status by monitoring the levels of pCBL in the same tissue samples using a customized CBL-pTyr371 antibody. In contrast to CIN85 and CBL, the level of pCBL was higher in the NAT and gradually decreased through the different stages of breast cancer until it became very low between stages II and III (Fig. 6e, f). This suggests that although there is a high level of CBL expression in advanced stages of breast cancer, its E3 ligase function is not activated. Under these circumstances, CBL could in principle act as an adaptor and recruit other proteins such as CIN85 to facilitate breast cancer progression. We, therefore, reasoned that disruption of the CBL–CIN85 interaction by PepC1 might reduce breast cancer cell proliferation. Since CBL and CIN85 show higher expression in MDA-MB-231 cells [43] (Supplementary Fig. 7), we treated these cells with PepC1 and found that the cells treated with PepC1 had a lower rate of proliferation as compared to cells treated with the cell-penetrating peptide Antennapedia (Cont) or PepC2 (Fig. 6g). These data suggest for a correlation between the protein levels of CIN85 and E3-inactivated CBL in facilitating breast cancer progression; hence, there might be therapeutic potential in disrupting the CBL–CIN85 interaction.

## Discussion

**Fig. 7:**
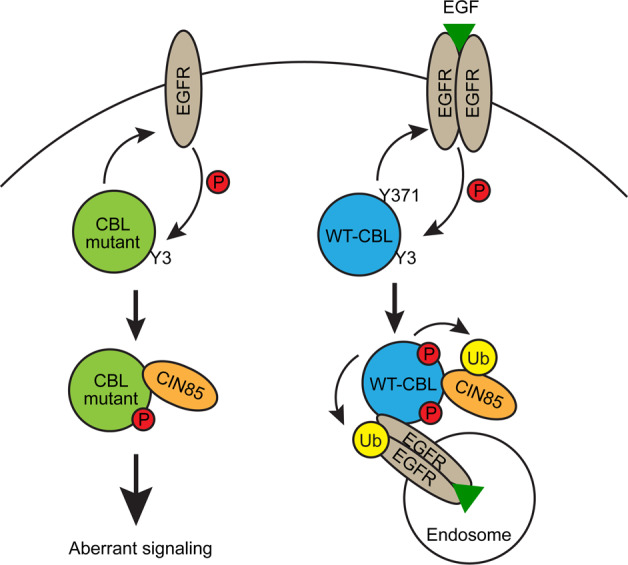
Model showing the mechanism of action of CBL mutant in promoting oncogenesis. RTKs like EGFR activates WT-CBL via Tyr371-phosphorylation. This ligase-activated CBL associates with adaptor proteins like CIN85 to promote subsequent endosomal sorting and lysosome-mediated degradation of EGFR. CBL Y371 mutants lack E3 ligase activity but still associate with EGFR and are phosphorylated within the C-terminal tyrosines (Y3) and are thus able to bind tightly to CIN85. These CBL mutant-CIN85 complexes have derailed endosomal trafficking associated with amplified signaling that promotes oncogenesis.

CBL’s dual functions as an E3 and an adaptor are tightly coupled to ensure proper regulation of RTK signaling. CBL mutations or deletions within the LHR and RING domain have been identified in a substantial fraction of different types of myeloid malignancies. A central feature of these CBL mutants is the loss of E3 activity, which decouples CBL’s dual functions, resulting in deregulation of adaptor proteins that activate various signaling pathways (Fig. 7). Our work provides a mechanistic link between the adaptor role of CBL mutants and CIN85 in eliciting oncogenesis. We showed that CBL mutants bind CIN85 in the absence of growth factor stimulation; this binding and the lack of E3 activity deregulates CIN85 trafficking and signaling. This results in alteration of the transcriptome landscape to promote various signaling events such as Cdc42 activation to drive oncogenic transformation. A coordinated event involving EGFR binding and subsequent EGFR-mediated phosphorylation of CBL mutants’ C-terminal tyrosines is essential for enabling interactions between an adjacent proline-rich motif and CIN85. In human breast cancer samples, we found a correlation between levels of CIN85 and E3-inactivated CBL. Furthermore, we identified a CIN85-binding peptide, PepC1, from the proline-rich region of CBL that reduces the proliferation of breast cancer cells. These results suggest that inhibiting interactions between mutant or E3-inactive CBL and RTKs or CIN85 in cancer patients could have therapeutic potential.

We showed that unlike the WT-CBL and CIN85 binding [2, 37]; CBL-Y371S binds CIN85 in the absence of growth factor stimulation. It seems that in the absence of receptor stimulation, there is basal EGFR activity as shown by EGFR-mediated phosphorylation of CBL mutants’ C-terminal tyrosines. However, why WT-CBL’s C-terminal tyrosines are not hyper-phosphorylated is unclear as EGFR is competent in phosphorylating the C-terminal Y731 of both WT-CBL and CBL mutants in vitro. We find that CBL-Y371S exhibits a slightly higher binding preference for EGFR as compared to WT-CBL, but this difference is moderate. As Tyr371 mutation induces LHR conformational change [50], we reason that altered CBL conformation could favor EGFR binding and EGFR-mediated C-terminal tyrosine phosphorylation. CBL-70Z lacks part of the LHR including Tyr371, suggesting it also disrupts LHR–TKBD interactions to alter CBL’s conformation. Although CBL-Y371F has not been identified as an MDS/MPN CBL mutant, previous studies showed that it lacks E3 activity but adopts a conformation similar to WT-CBL [50]. We showed that this mutant binds CIN85 albeit not to the same extent as MDS/MPN CBL Y371 mutants. Moreover, we found that other MDS/MPN CBL mutants located in the RING domain rather than the LHR, such as R420Q and R420L, disrupt E2~Ub binding but still bind strongly to CIN85 in the absence of growth factor stimulation. Thus, the lack of E3 activity contributes to the enhanced CIN85 binding, and mutation-induced conformational changes might facilitate the process.

Several groups have independently demonstrated that the TKBD, proline-rich regions, and C-terminal tyrosines are important for CBL mutants to elicit oncogenic potential [22, 30, 31]. We showed that these domains function in a coordinated manner in which the TKBD is critical for initial binding to EGFR to allow EGFR-mediated phosphorylation of C-terminal tyrosines of CBL mutants and subsequent CIN85 binding to a C-terminal proline-rich motif in CBL mutants. Our mass spectrometry analyses also identified GRB2 and CD2AP that bind CBL-70Z in the absence of growth factor stimulation. We speculate that they might contribute to aberrant CBL mutant signaling depending on the cell system. A common theme for the oncogenic potential of CBL mutants is the activation of select downstream signaling pathways including PI3K-AKT, STATs, and ERK [22, 27, 28, 31, 32]. Understanding CBL mutant interactomes offers the opportunity to block the initiation of these aberrant signaling cascades. Indeed we showed that knockdown of CIN85, disruption of the CIN85-binding mechanism, or disruption of the RTK–CBL interaction, reduced the transforming ability of CBL-Y371S, suggesting that inhibiting these events might have therapeutic potential.

CBL–CIN85 complex is assembled upon growth factor stimulation and localizes to endosomal vesicles to facilitate lysosomal degradation of RTKs [2, 3]. It is unclear what the consequences of failure are in sorting of CIN85 to the endosomes. We showed that E3-inactive CBL mutants were unable to ubiquitinate and traffic CIN85 to the endosomes. This would deregulate RTK signaling where RTKs like EFGR could act as transcription factors [51] or activate additional transcription factors via signaling proteins like AKT, thereby altering the transcriptome landscape. CIN85 binds many proteins via its SH3 domains and proline-rich region [52]. It seems likely that CBL mutant-CIN85 complex could recruit other signaling proteins to form large scaffolding complexes and activate transcriptional events. Our results showed that CBL mutant-CIN85 complexes alter the transcriptome and consequently activate Cdc42 to modulate AKT pathway. This process is independent of PI3K and we posit that transcriptional upregulation of Fgd4 could contribute to Cdc42 activation.

Several studies have shown that CBL–CIN85 complex interacts with a number of proteins to promote cancer progression [34, 43, 48, 49]. AMAP1 localizes with CBL–CIN85 complex at invadopodia to advance breast cancer cell invasion [43] and MUC1 association with CBL–CIN85 complex at the plasma membrane and in the cytosol contributes to the progression of colon cancer [34]. How WT-CBL is deregulated, however, remains unclear. We found a stage-wise increase in the expression levels of both CIN85 and CBL in breast cancer samples. In contrast, levels of pCBL were high in cancer adjacent normal breast tissue and considerably low in advanced stages of cancer. This inverse relationship between CBL expression and its Tyr371-phosphorylation status implies that in the advanced stages of breast cancer the high levels of CBL remain in an E3-inactivated state in which CBL mainly functions as an adaptor. It seems plausible that E3-inactivated CBL could function in a similar manner to the MDS/MPN CBL mutants and cooperate with CIN85 to promote cancer progression. Indeed, knocking down CBL in different cancer cells reduces their proliferation, clonogenic survival, and migration [43, 53]. It is noteworthy that none of these studies show the activation status of CBL. Nonetheless, we find the reduced proliferation of MDA-MB-231 cells when treated with the PepC1 peptide that blocks the interaction between CIN85 and CBL. Although PepC1 binds CIN85 with a *K*_d_ of 5.5 μM, it remains unclear whether PepC1 could interact with other protein(s) in cells or the chemical property of PepC1 could contribute to the reduced cell proliferation. Future optimization of PepC1 is required to improve its specificity and efficacy. Likewise screening for peptides targeting additional CBL domains would be beneficial in targeting CBL in CBL-dependent cancer.

In summary, our results elucidate how MDS/MPN CBL mutants or inactivated CBL elicit gain-of-function properties by deregulating CBL–CIN85 signaling and provide evidence that targeting the RTK–CBL–CIN85 axis could inhibit oncogenesis.

## Materials and methods

Detailed methods including plasmids, antibodies, shRNA knockdown, mass spectrometry and RNAseq analyses, ubiquitination and kinase assays, cDNA synthesis and quantitative real-time PCR, Cdc42 activation assay, recombinant protein preparation, SPR analyses, breast cancer tissue microarray, cell proliferation assays are provided in the Supplemental Information.

### Cell maintenance and transfections

Mammalian cell lines HEK293, MDA-MB-231, and NIH-3T3 were obtained from ATCC. The HeLa cell line was procured from EACC. HEK293 and NIH-3T3 cells were cultured in DMEM; MDA-MB-231 cells were cultured in DMEM/F12 and HeLa cells were cultured in RPMI. The cell lines were grown in a monolayer at 37 °C in 5% CO_2_, supplemented with 20 mM l-glutamine, 6 mg/l gentamycin, 0.1 mg/ml streptomycin, and 100 units/ml penicillin (Invitrogen, USA). All cell lines except NIH-3T3 (supplemented with 10% DBS) were also supplemented with 10% FBS. Short tandem repeat profiling using GenePrint 10 System (Promega) is used to authenticate the cell lines in-house every 2 years. Cell lines are also regularly tested in-house for mycoplasma. Lipofectamine-2000 (ThermoFisher Scientific) or jetPEI DNA transfection reagent (Polyplus transfection) reagents were used to transfect the cell lines with the plasmids following the manufacturer’s protocol. Unless otherwise mentioned, cells were harvested 48 h after transfection.

### Immunoblotting and co-immunoprecipitation

Cells were resuspended in IP lysis buffer (50 mM Tris-HCl pH 7.5, 150 mM NaCl, 1 mM EDTA, 1% IGEPAL CA-630, 10% glycerol, 0.5 mM DTT, and cOmplete protease inhibitor cocktail) to prepare whole cell lysates for western blot and immunoprecipitation as described previously [54]. For standard immunoprecipitations, 1 mg of freshly prepared whole-cell lysates were precleared by incubating them with 25 μl (50% slurry) of a 1:3 mixture of Protein G Sepharose 4 Fast Flow and Protein A Sepharose CL 4B beads on a rotatory shaker at 4 °C for 30 min. The indicated antibodies were then added to the collected supernatants and incubated overnight on a rotatory shaker at 4 °C. The following day, samples were further incubated for 2 h at 4 °C after adding 35 μl (50% slurry) beads. For immunoprecipitation using Myc-trap, the lysates were only incubated for 1 h with the trap beads. The beads were washed twice with IP lysis buffer and once with IP wash buffer (IP lysis buffer consisting of 200 mM NaCl and 1.0 mM DTT). Immunoprecipitated proteins were eluted in 40 μl 2× loading dye at 95 °C. Immunoblotting was performed as described previously [54] and the immunoblots were scanned on an Odyssey CLx Imaging System (LI-COR Biosciences).

### Immunocytochemistry

The cells on coverslips were washed thrice with PBS, fixed in methanol, and blocked with 3% BSA in PBS. Primary antibodies in 1% BSA were added and the coverslips were put in a moist chamber for overnight incubation at 4 °C. This was followed by incubation with secondary antibodies for 1 h at room temperature under dark conditions and the coverslips were then mounted onto glass slides containing DAPI media. The images were captured on a Zeiss 710 upright confocal microscope at ×60 (oil) magnification.

### Foci-formation assay

NIH-3T3 cells were seeded onto 6 cm dishes, followed by two successive rounds of infections with the respective constructs as described previously [50]. The cells were selected with 1.5 μg/ml puromycin (Sigma-Aldrich), cultured for 18 days, fixed in methanol, and stained with sulforhodamine B (Sigma-Aldrich). Cells transfected with KRAS-G12D were cultured for 7 days. Three independent experiments were performed. Foci were then counted manually and statistical analyses were performed with Prism (Graph Pad, version 8.1.0).

### Quantification and statistical analysis

Statistical analyses were performed with GraphPad Prism (version 8.1.0). Student’s t-tests (two-tailed) were performed to analyze statistical significance with p-values <0.05 accepted as statistically significant. The error bars present in the foci-formation assay figures represent ± SEM. Representative immunoblots and immunocytochemistry images were shown and the experiments were performed in triplicate unless otherwise mentioned. For immunoblot band quantification, intensities were measured using Fiji as detailed in the figure legends. For SPR assays, Biacore T200 BIAevaluation software (GE Healthcare) was used for the analyses of data by steady-state affinity. The number of replicates (*n*) for each *K*_d_ measurement is indicated in Supplementary Fig. S2. The bar graphs for relative expression in quantitative real-time PCR represent 95% confidence intervals. GraphPad Prism (version 8.1.0) was used to generate the plots in Figs. 4c, g, 6b, d, f, g and Supplementary Figs. 1c, 3a, b, c, e, and the error bars represent ± SEM.

## Supplementary information

Supplemental Material

## Data Availability

Raw mass spectrometry proteomics data have been deposited to the ProteomeXchange Consortium (http://proteomecentral.proteomexchange.org) via the PRIDE partner repository with the data set identifier PXD011369. Gene expression profiling data based on RNAseq for WT-CBL, CBL-70Z, and CBL-Y371S have been deposited in the Gene Expression Ominibus (GEO) under accession code GSE121176. Uncropped immunoblots are provided in Supplementary Fig. 8. All data generated in this study are included in this article (and its supplementary files) or are available from the corresponding author upon request.
