# Supplementary material for: E3 ligase-inactivation rewires CBL interactome to elicit oncogenesis by hijacking RTK–CBL–CIN85 axis
Source: Oncogene. 2021 Feb 24;40(12):2149–64. doi: 10.1038/s41388-021-01684-x (PMC7994203; doi:10.1038/s41388-021-01684-x)
Supplement: Supplementary file 1 — Supplemental Material [file 41388_2021_1684_MOESM1_ESM.docx]

**Supplementary Information**

**E3 ligase-inactivation rewires CBL interactome to elicit oncogenesis by hijacking RTK-CBL-CIN85 axis**

Syed Feroj Ahmed, Lori Buetow, Mads Gabrielsen, Sergio Lilla, Gary J. Sibbet, David Sumpton, Sara Zanivan, Ann Hedley, William Clark, and Danny T. Huang

**
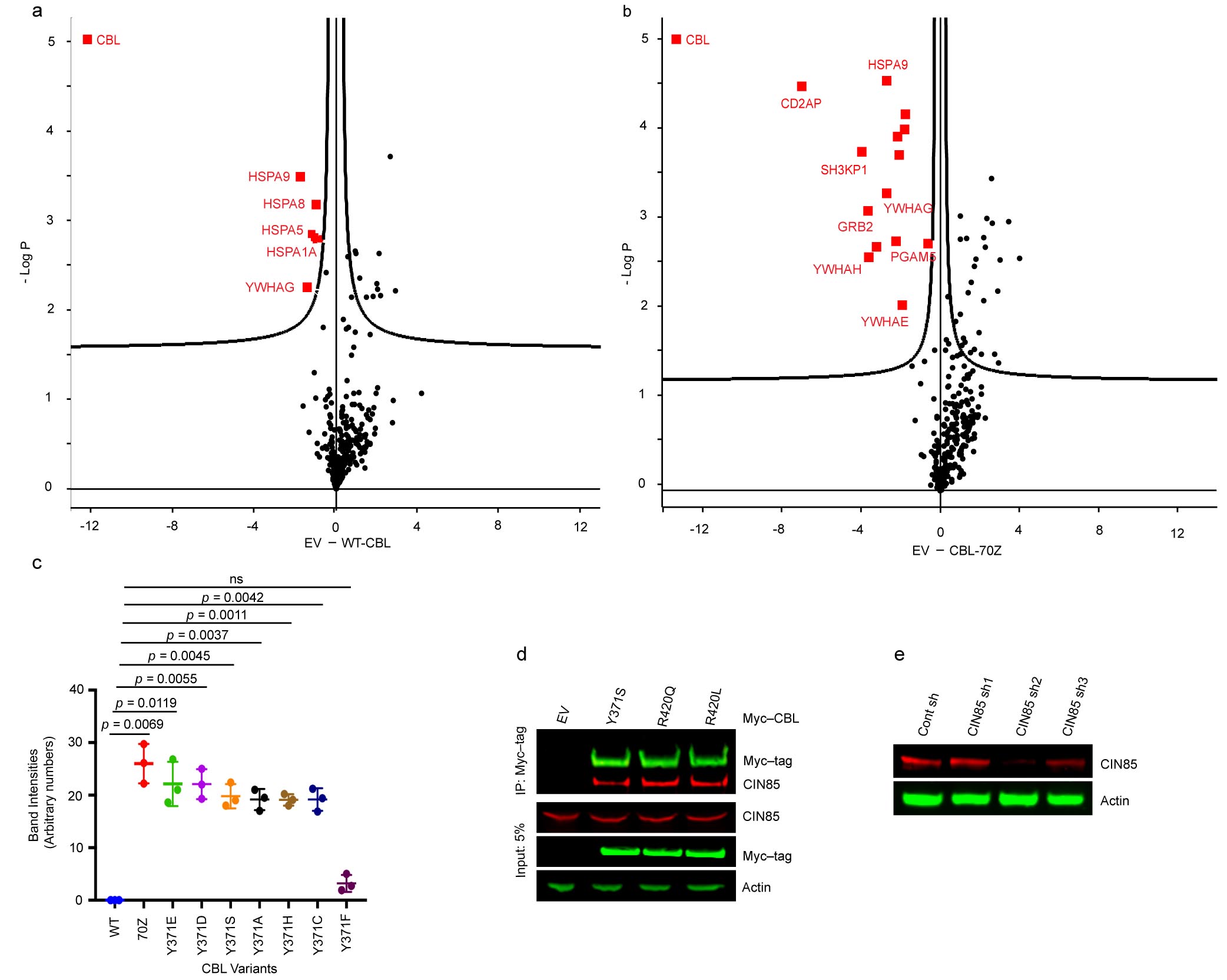
**

**Supplementary Fig. 1: WT-CBL and CBL-70Z show differences in interactomes, related to Figure 1. a** Volcano plot showing differences in interactomes of empty vector (EV) and WT-CBL. Experiment was performed in triplicate. **b** Volcano plot showing differences in interactomes of empty vector (EV) and CBL-70Z. Experiment was performed in triplicate. **c** Quantification of immunoprecipitated CIN85 band intensities in Fig. 1c. Band intensities of immunoprecipitated CIN85 against each CBL variant were quantified and the values from (n = 3) independent experiments were plotted. Error bars represent ± SEM and Student’s t-test was used to calculate p-values**. d** Co-immunoprecipitation assay of HEK293 cell lysates with overexpressed Myc-tagged CBL-Y371S, CBL-R420Q, CBL-R420L or the EV. Anti-Myc immunoprecipitates and cell lysates were analyzed by immunoblotting with anti-CIN85, anti-Myc tag and anti-actin (loading control) antibodies as indicated. **e** Knock down efficiency of CIN85 shRNAs. Lysates from HEK293 cells transfected with Cont sh, CIN85 sh1, CIN85 sh2 and CIN85 sh3 were analysed by immunoblotting with anti-CIN85 and anti-actin antibodies.

**
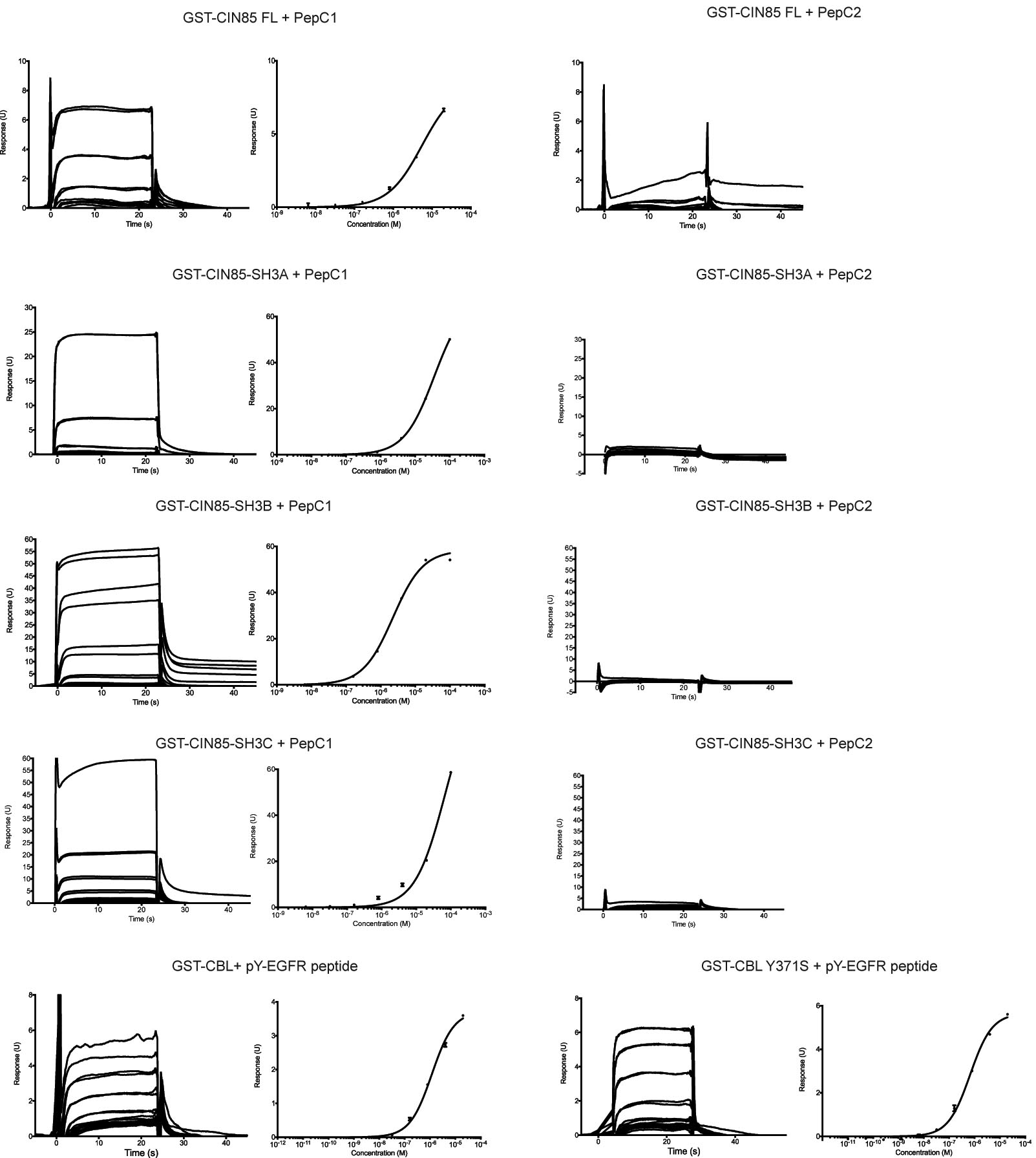
**

**Supplementary Fig. 2: SPR analyses of GST-bound ligand and analyte binding affinities, related to Table 1.**

Representative sensorgrams (left) and binding curves (right) for GST-CIN85 FL, GST-CIN85-SH3A, GST-CIN85-SH3B, GST-CIN85-SH3C, GST-WT-CBL (47-435) or GST-CBL-Y371S (47-435) and the analytes PepC1, PepC2 or pY-EGFR peptide as indicated above each sensorgram and binding curve set. Replicate (n = 2) for each binding curve. PepC2 did not bind CIN85 and therefore binding curves were not shown.

**
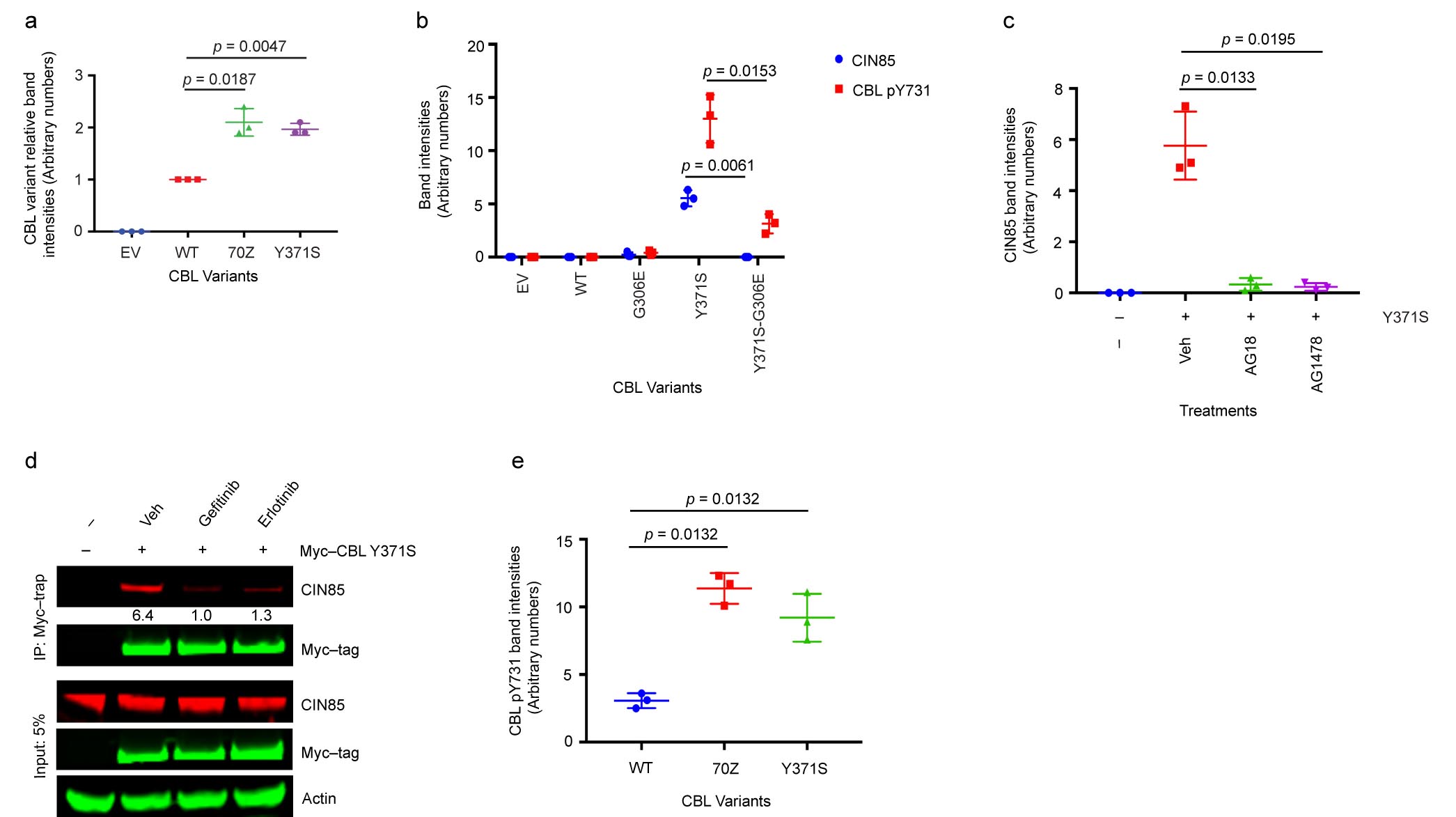
**

**Supplementary Fig. 3: CBL-Y371S requires RTK binding for its oncogenic potential. a** Quantification of immunoprecipitated CBL variant band intensities in Fig 3a. Band intensities of immunoprecipitated CBL variants were quantified and the values from (n = 3) independent experiments were plotted. **b** Quantification of immunoprecipitated CIN85 and CBL pY731 band intensities in Fig 3b. Band intensities of immunoprecipitated CIN85 and CBL pY731 against each CBL variant were quantified and the values from (n = 3) independent experiments were plotted. **c** Quantification of immunoprecipitated CIN85 band intensities in Fig 3c. Band intensities of immunoprecipitated CIN85 against CBL Y371S following different treatments were quantified and the values from (n = 3) independent experiments were plotted. **d** Co-immunoprecipitation assay of HEK293 cell lysates with overexpressed Myc-tagged CBL-Y371S or EV, left untreated or treated with vehicle (Veh), 10 μM Gefitinib or 20 μM Erlotinib for 24 h. Anti-Myc immunoprecipitates and cell lysates were analyzed by immunoblotting with anti-CIN85, anti-Myc tag and anti-actin antibodies as indicated. Band intensities of immunoprecipitated CIN85 are quantified and average values from (n = 2) independent experiments relative to Y371S (Gefitinib treated) are represented below the bands. **e** Quantification of CBL variant pY731 band intensities in Fig 3d. Band intensities of pY731 of each CBL variant were quantified and the values from (n = 3) independent experiments were plotted. Error bars represent ± SEM and Student’s t-test was used to calculate p-values in **a**, **b**, **c** and **e.**

**
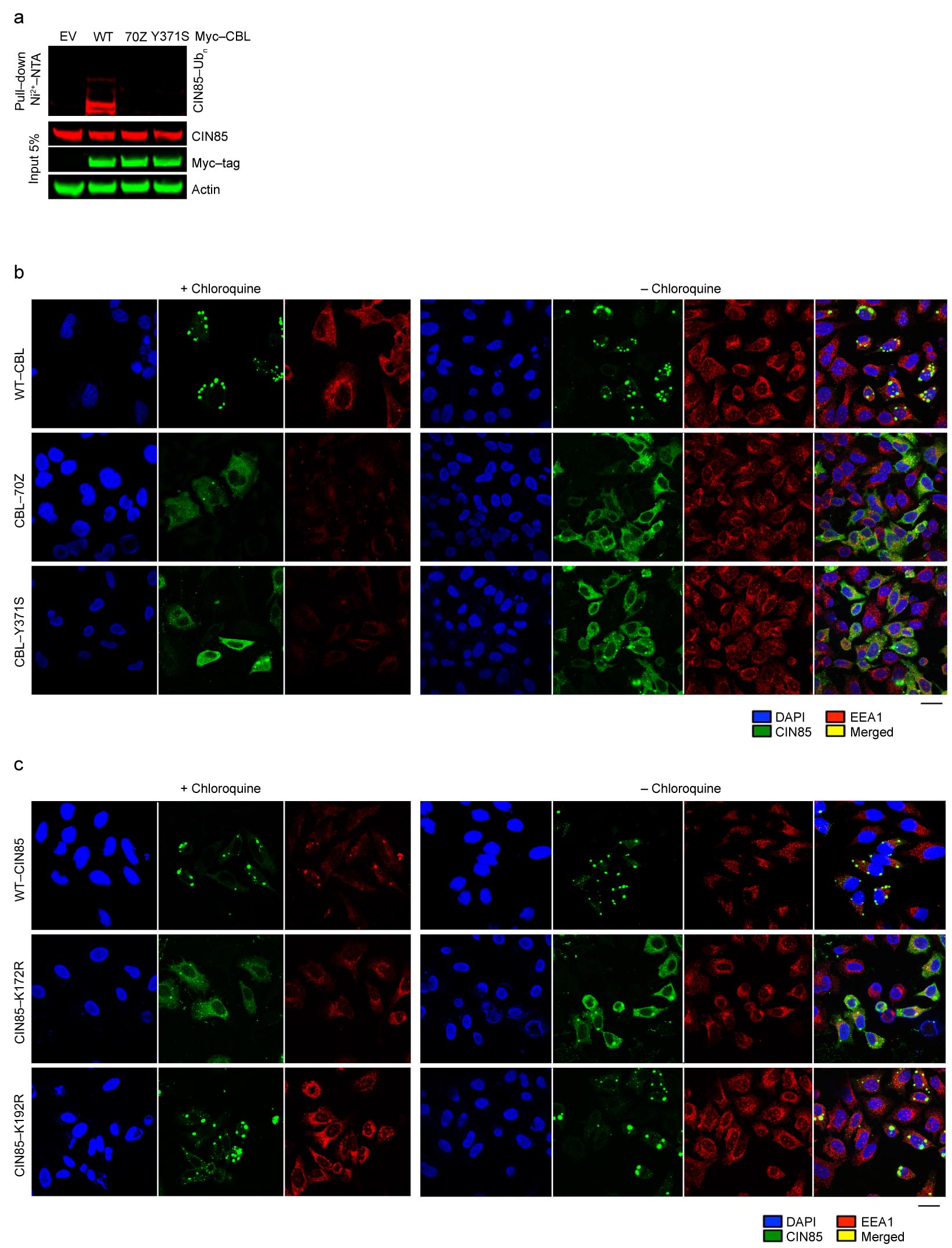
**

**Supplementary Fig. 4: CBL mutants fail to ubiquitinate and traffick CIN85 to endosomes, related to Fig. 4.** **a** Ubiquitination assay of HEK293 cell lysates with overexpressed Myc-tagged WT-CBL, CBL-70Z, CBL-Y371S, or EV along with His-Ub. Ni^2+^–pull-down products and the cell lysates were analyzed by immunoblotting against anti-CIN85, anti-Myc tag and anti-actin antibodies as indicated; n = 3 independent experiments. **b** Individual images related to Fig. 4b (chloroquine treated; left) and chloroquine untreated (right). HeLa cells stably expressing WT-CBL, CBL-70Z or CBL-Y371S were overexpressed with GFP-tagged CIN85 (green), fixed and subjected to immunofluorescence staining using antibodies against EEA1 (red) and DAPI (blue, nuclei). **c** Individual images related to Fig. 4f (chloroquine treated; left) and chloroquine untreated (right). HeLa cells stably expressing WT-CBL were overexpressed with GFP-tagged WT-CIN85, CIN85-K172R or CIN85-K192R (all green), fixed and subjected to immunofluorescence staining using antibodies against EEA1 (red) and DAPI (blue, nuclei). Images were captured using confocal microscopy. Scale bars, 25μm. The cells were treated with 50 μM chloroquine or left untreated for 24 hours and stimulated with 50 ng/ml EGF for 5 min prior to processing.

**
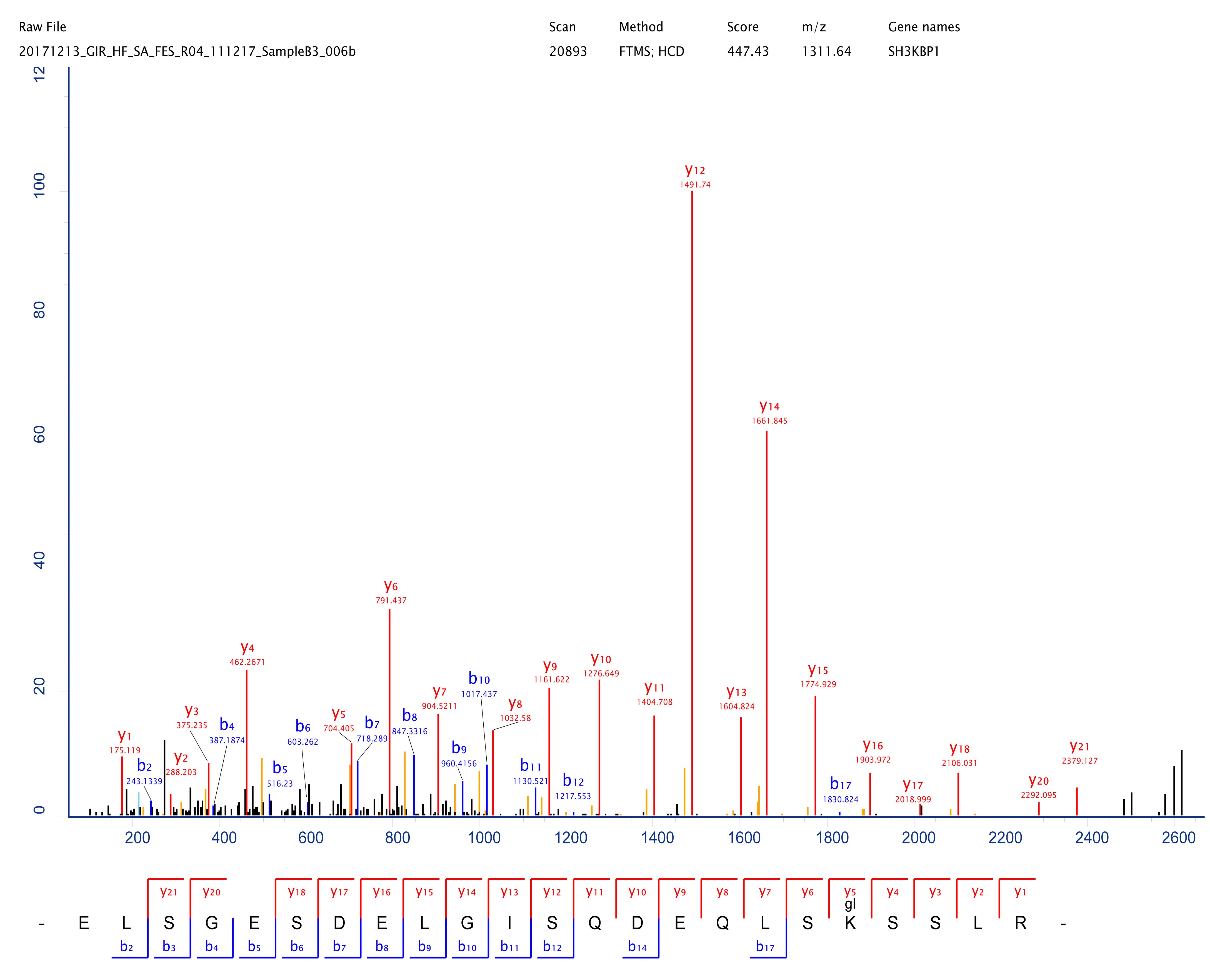
**

**Supplementary Fig. 5: Mass spectra profile for ubiquitinated CIN85-K172, related to Fig. 4d.**

**
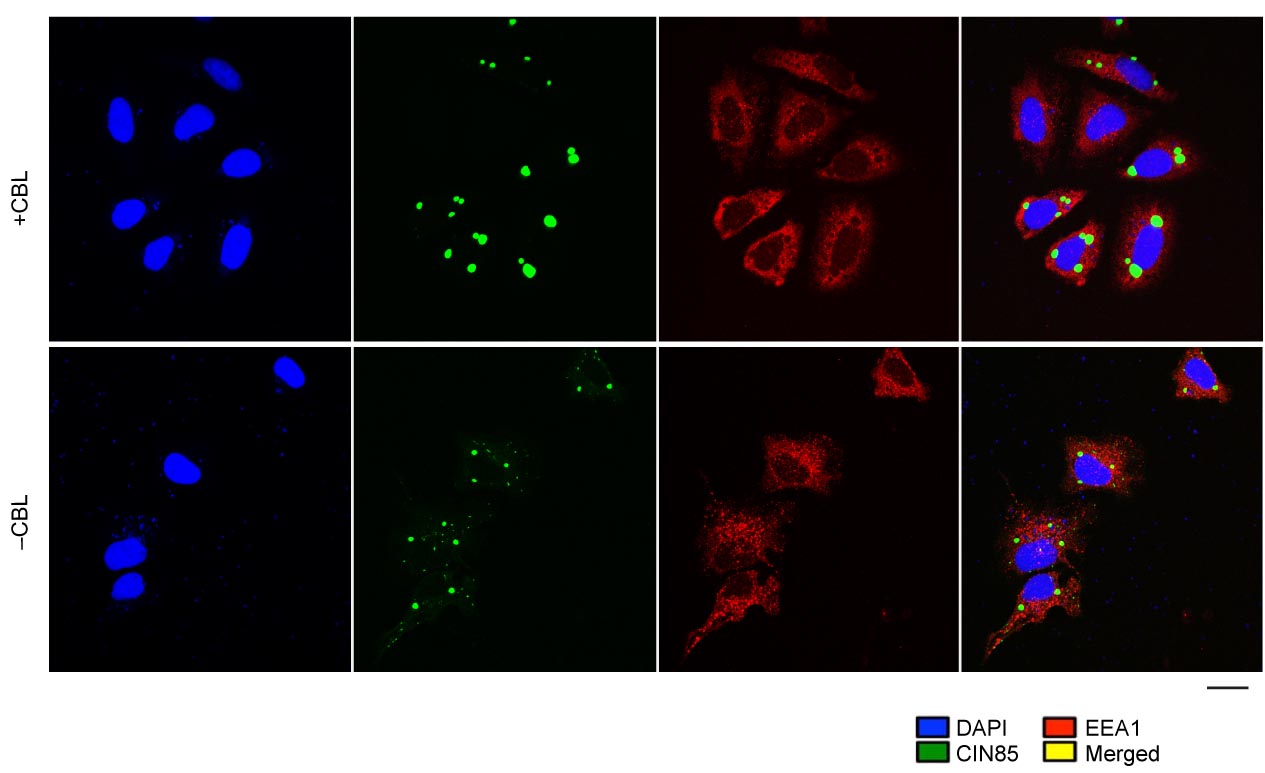
**

**Supplementary Fig. 6:** **WT-CBL overexpression induces fusion of early endosomes, related to Fig. 4.** HeLa cells stably expressing or not expressing WT-CBL were overexpressed with GFP-tagged CIN85 (green), fixed and subjected to immunofluorescence staining using antibodies against EEA1 (red) and DAPI (blue, nuclei). Images were captured using confocal microscopy. Scale bars, 25μm. The cells were treated with 50 μM chloroquine for 24 hours and stimulated with 50 ng/ml EGF for 5 min prior to processing.

**
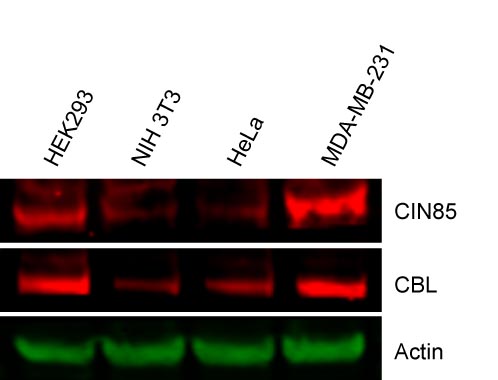
**

**Supplementary Fig. 7:** **Endogenous expression levels of** **CBL and CIN85 in different cell lines.** Lysates from mammalian cell lines HEK293, NIH3T3, HeLa and MDA-MB-231 were analyzed by immunoblotting against anti-CIN85, anti-CBL and anti-actin antibodies as indicated.

**
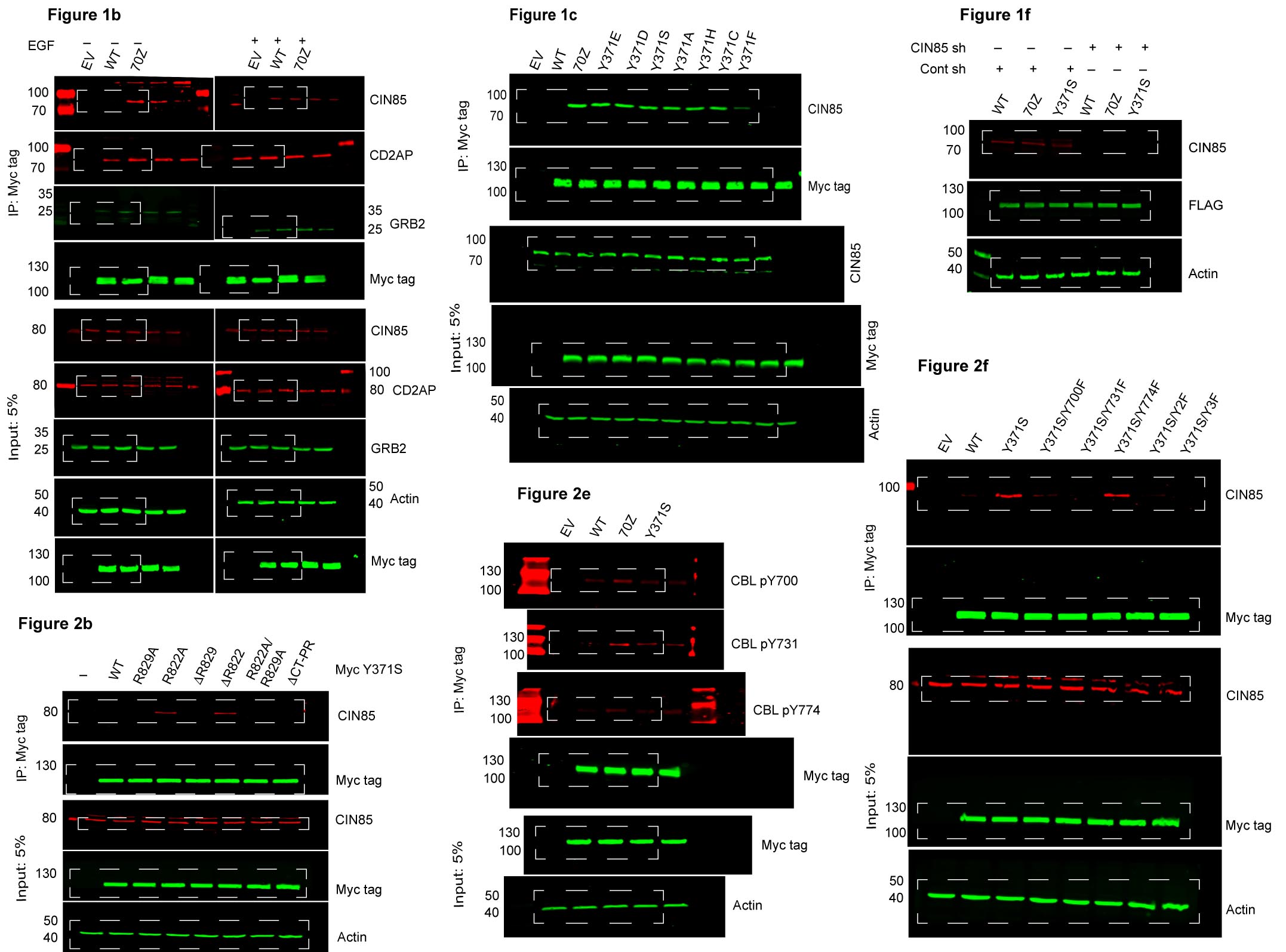
**

**
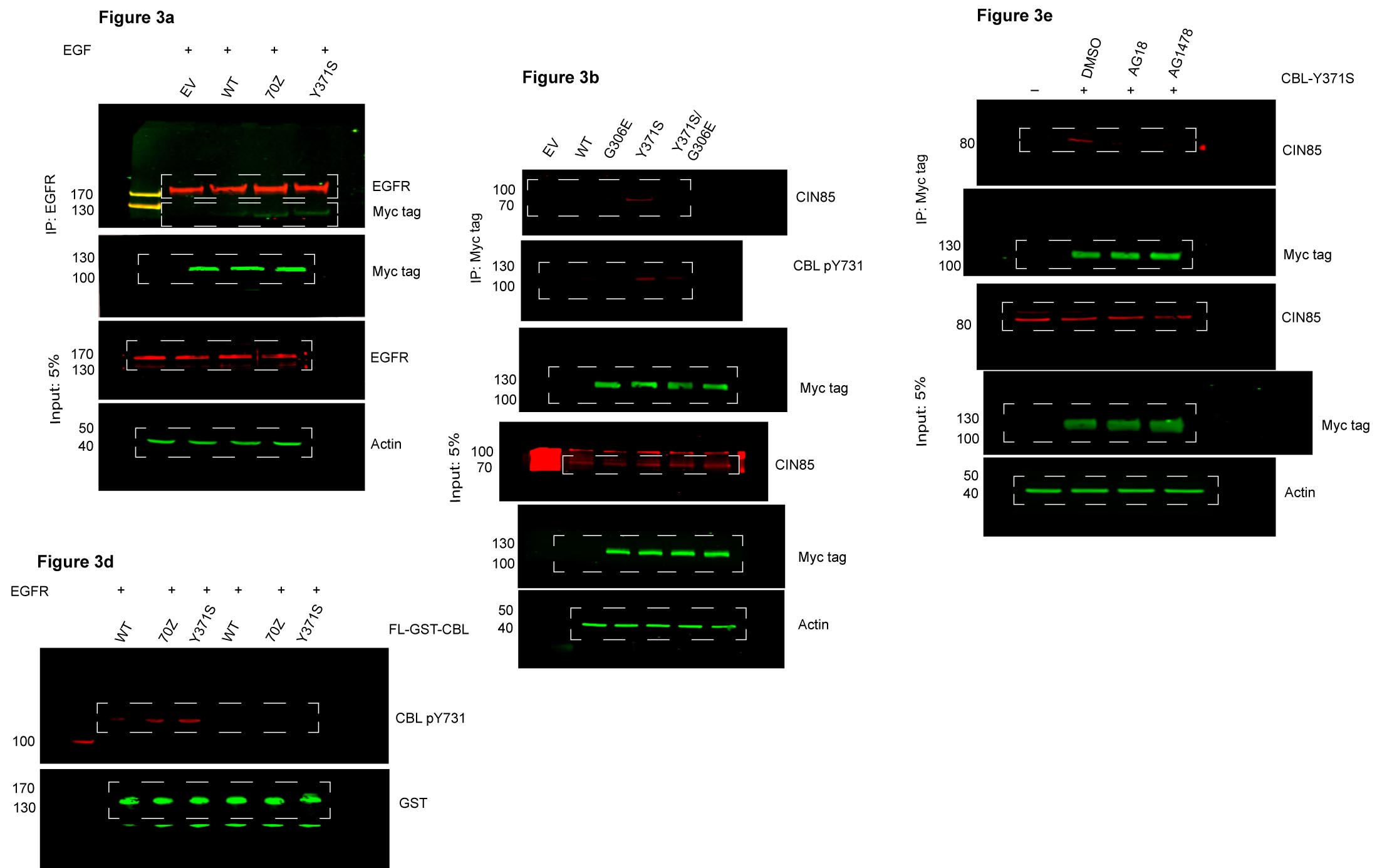
**

**
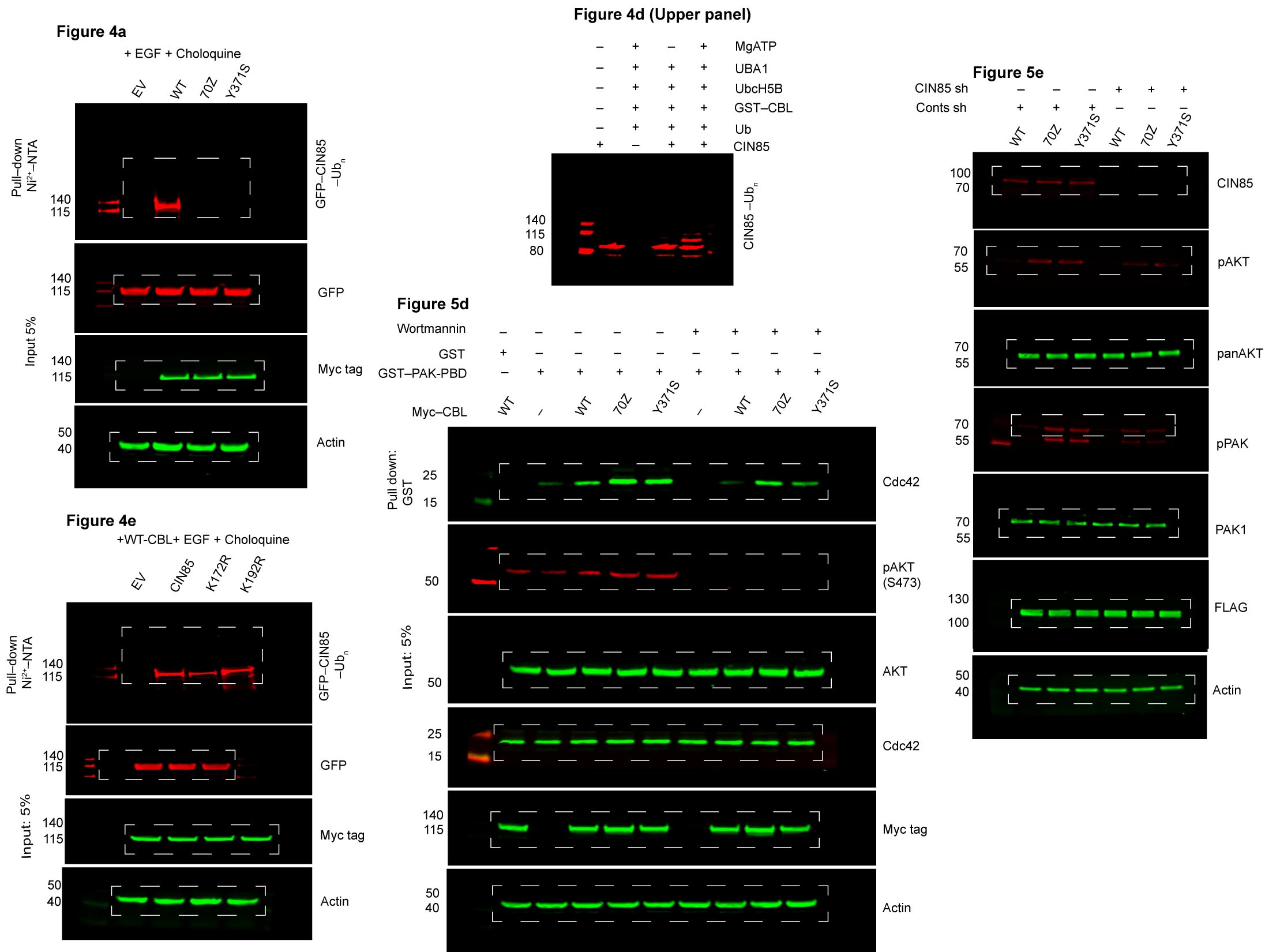
**

**
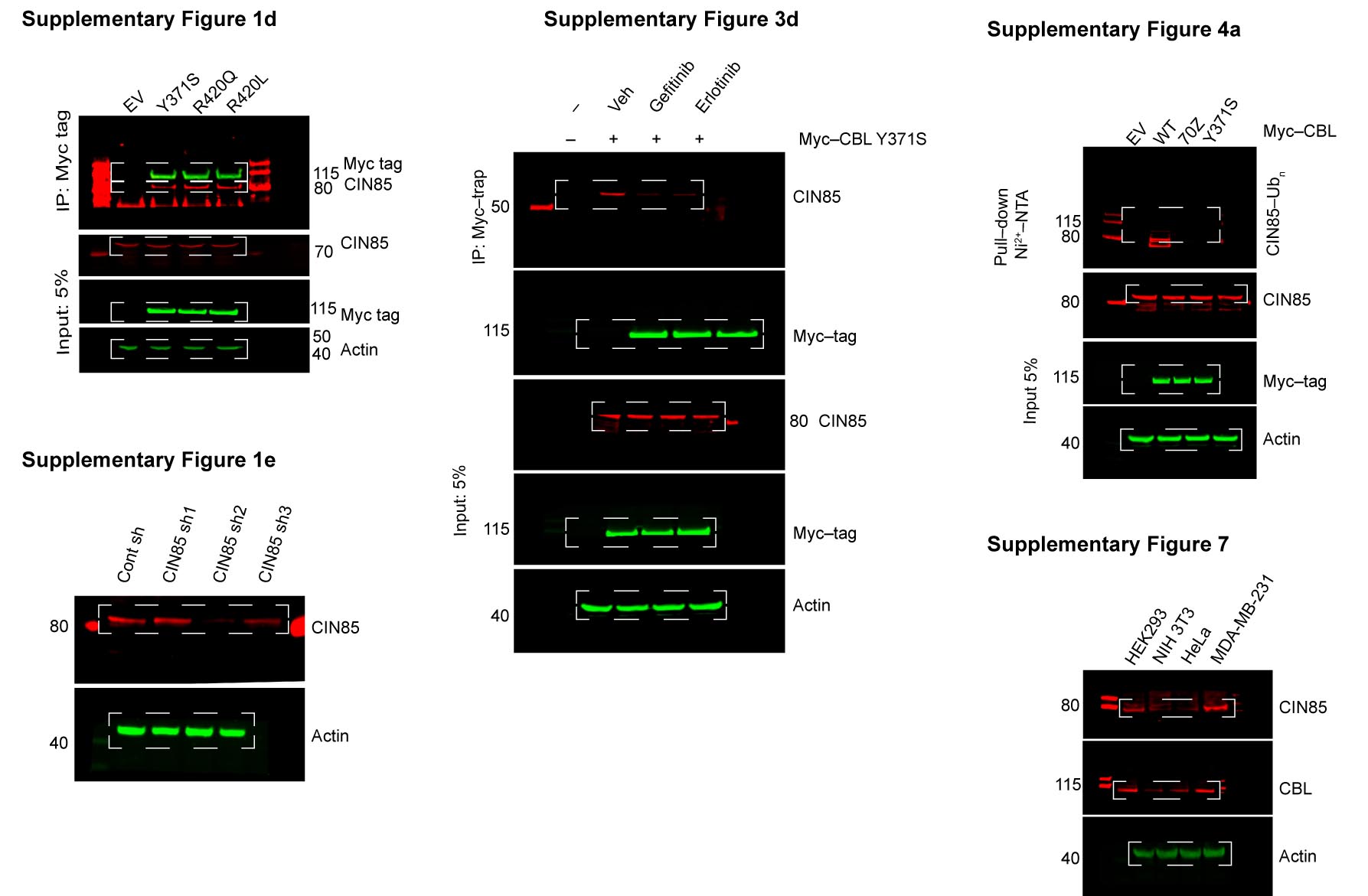
**

**Supplementary Fig. 8:** Unprocessed images of immunoblots. Unprocessed images of scanned immunoblots as shown in Figures and Supplementary Figures are provided. Regions that are presented in the main figures are outlined with a dashed grey rectangle.

**Materials and Methods**

Plasmids

The following constructs were generated in a previous study [50]: pcDNA3.1 Myc-CBL, pcDNA3.1 Myc-CBL-70Z, pcDNA3.1 Myc-CBL-Y371E, pcDNA3.1 Myc-CBL-Y371D, pcDNA3.1 Myc-CBL-Y371S, pcDNA3.1 Myc-CBL-Y371A, pcDNA3.1 Myc-CBL-Y371H, pcDNA3.1 Myc-CBL-Y371C, pcDNA3.1 Myc-CBL-Y371F, pBabe Puro FLAG-CBL, pBabe Puro FLAG-CBL-70Z, pBabe Puro FLAG-CBL-Y371S; pET23d *Arabidopsis thaliana* Uba1, pRSF_1b UbcH5B and RSF_Duet His-TEV GGS-Ub were designed as described previously [7].

The following constructs were generated in this study: pLKO.1 Cont sh, pLKO.1 CIN85 sh1, pLKO.1 sh2 (CIN85 sh), pLKO.1 sh3, pBabe Puro KRAS-G12D, pcDNA3.1 Myc-CBL-R420Q, pcDNA3.1 Myc-CBL-R420L, pcDNA3.1 Myc-CBL-Y371S/R829A, pcDNA3.1 Myc-CBL-Y371S/R822A, pcDNA3.1 Myc-CBL-Y371S/ΔR829A, pcDNA3.1 Myc-CBL-Y371S/ΔR822A, pcDNA3.1 Myc-CBL-Y371S/R822A/R829A, pcDNA3.1 Myc-CBL-Y371S/ΔCT-PR (Δ822-830), pBabe Puro CBL-Y371S/R822A, pBabe Puro CBL-Y371S/R829A, pBabe Puro CBL-Y371S/R822A/R829A, pcDNA3.1 Myc-CBL-Y371S/Y700F, pcDNA3.1 Myc-CBL-Y371S/Y731F, pcDNA3.1 Myc-CBL-Y371S/Y774F, pcDNA3.1 Myc-CBL-Y371S/Y700F/Y731F (Y2F), pcDNA3.1 Myc-CBL-Y371S/Y700F/Y731F/Y774F (Y3F), pBabe Puro FLAG-CBL-Y371S/Y700F, pBabe Puro FLAG-CBL-Y371S/Y731F, pBabe Puro FLAG-CBL-Y371S/Y774F, pBabe Puro FLAG-CBL-Y371S/Y3F, pcDNA3.1 Myc-CBL-G306E, pcDNA3.1 Myc-CBL-Y371S/G306E, pcDNA3.1 12X His-Ub, pGZ21dxZ GFP-CIN85, pGZ21dxZ GFP-CIN85-K172R, pGZ21dxZ GFP-CIN85-K192R, pGEX4T1 GST-TEV CIN85 FL, pGEX4T1 GST-TEV CIN85-SH3A (1-58), pGEX4T1 GST-TEV CIN85-SH3B (98-157), pGEX4T1-TEV CIN85-SH3C (263-328), pGEX4T1 His-GST-TEV CBL FL, pGEX4T1 His-GST-TEV CBL-70Z FL, pGEX4T1 His-GST-TEV CBL-Y371S FL, pGEX4T1 GST-TEV CBL (47-435) and pGEX4T1 GST-TEV CBL-Y371S (47-435).

shRNA knock down and stable cell line preparation

Target sequences for human specific CIN85-shRNA1 (5′–GAAAGTTGGCGACATC

ATAGA–3′), CIN85-shRNA2 (5′–AATGACATTGACTTAGAAGGT–3′) and CIN85-shRNA3 (5′–AAGAGGATAAGGAGGAACACA–3′) were selected using Whitehead Institute siRNA designing tool. Following a previously described protocol [I], sense and antisense oligonucleotides synthesized by IDT (Integrated DNA Technologies, Coralville, IA, USA) were annealed and inserted into pLKO.1 expression vector (Addgene). CIN85-shRNA2, which showed the best CIN85 knockdown efficiency (Supplementary Fig. 1e), was used to perform the subsequent experiments. Control shRNA in pLKO.1 was procured from Addgene. For stable cell line preparation, the human cell lines were transfected with the desired construct, selected and maintained in 1.5 μg/ml puromycin. Alternatively, Phoenix Eco cells were transiently transfected with the desired constructs and the packaged viral particles were used to infect NIH-3T3 cells (mouse fibroblast) followed by puromycin selection (2.0 μg/mL) as described previously [50].

Antibodies, chemicals and kits

Primary antibodies used in the study include: mouse anti-Myc tag (Cell Signaling, cat. no. 2276, 1:1,000 for western blot and 1:500 for immunoprecipitation), rabbit anti-CIN85 (Cell Signaling, cat. no. 12304, 1:1,000 for western blot), rabbit anti-CIN85 (Abcam, cat. no. ab151574, 1:200 for immunohistochemistry), goat anti-Actin (Santa Cruz Biotechnology, cat. no. sc-1616, 1:1,000 for western blot), mouse anti-CD2AP (Santa Cruz, cat. no. sc-25272, 1:1,000 for western blot), rabbit anti-GRB2 (Santa Cruz Biotechnology, cat. no. sc-225, 1:1,000 for western blot), rabbit anti-FLAG (Cell Signaling, cat. no. 14793, 1:1,000 for western blot), rabbit anti-phospho-CBL-Y700 (Cell Signaling, cat. no. 8869, 1:1,000 for western blot), rabbit anti-phospho-CBL-Y731 (Cell Signaling, cat. no. 3554, 1:1,000 for western blot), rabbit anti-phospho-CBL-Y774 (Cell Signaling, cat. no. 3555, 1:1,000 for western blot), rabbit anti-CBL (Santa Cruz Biotechnology, cat. no. sc-170, 1:200 for immunohistochemistry), rabbit anti-CBL-pY371 antibody was generated by Eurogentec (1:200 for immunohistochemistry), goat anti-GST (GE Healthcare, cat. no. 27457701v, 1:1,000 for western blot), rabbit anti-EGFR (Merck Millipore, cat. no. 06-847, 1:1,000 for western blot and 1:500 for immunoprecipitation), rabbit anti-EEA1 (Cell Signaling, cat. no. 3288, 1:100 for immunofluorescence), rabbit anti-phospho-AKT-Ser473 (Cell Signaling, cat. no. 9271, 1:1,000 for western blot), mouse anti-AKT (Cell Signaling, cat. no. 2920, 1:1,000 for western blot), rabbit-phospho-PAK1/PAK2/PAK3 (Abcam, cat. no. 40795, 1:1,000 for western blot), PAK1 (Abcam, cat. no. 154284, 1:1,000 for western blot), mouse anti-GFP (Santa Cruz Biotechnology, cat. no. sc-81045, 1:1000 for western blot). The following secondary antibodies were used: goat anti-rabbit IRDye 680LT (LI-COR Biosciences, cat. no. 925-68021, 1:20,000 for western blot), goat anti-mouse IRDye 680LT (LI-COR Biosciences, cat. no. 925-68020, 1:20,000 for western blot), goat anti-rabbit IRDye 800CW (LI-COR Biosciences, cat. no. 925-32211, 1:15,000 for western blot), goat anti-mouse IRDye 800CW (LI-COR Biosciences, cat. no. 925-32210, 1:15,000 for western blot), donkey anti-goat IRDye 800CW (LI-COR Biosciences, cat. no. 925-32214, 1:15,000 for western blot) and goat anti-rabbit Alexa Fluor 594 (ThermoFisher Scientific, cat. no. A11012, 1:250 for immunofluorescence). The sources of the compounds and kits are as follows: AG18 and AG1478 (Merck Millipore), Gefitinib, Erlotinib, Wortmannin and hEGF (Sigma–Aldrich), ATP (Fisher Bioreagents), chloroquine and active Cdc42 detection kit (Cell Signaling Technology). DAPI containing medium was obtained from Vectashield while Myc-trap was purchased from Chromotek.

Mass Spectrometry

The empty vector (EV), full-length WT-CBL and CBL-70Z were expressed in HEK293 cells and immunoprecipitated with Myc-trap in triplicate experiments. The beads were washed twice with IP lysis buffer and once each with IP wash buffer and HPLC grade water, and processed following a previously established protocol [II]. Digestion of samples was performed by subsequent use of both endoLysC and trypsin in AMBIC. The separation of digested immunoprecipitated proteins, acquisition and processing of raw data were done as described previously [III] with some modifications. Searching of the database was done assuming trypsin cleavage with two allowed missed cleavages. Cysteine carbamidomethylation was specified as a fixed modification while N-terminal acetylation, methionine oxidation and “GlyGly” on Lysine (Ubiquitination) were set as variable modifications. The peptide, protein and site false discovery rate (FDR) was set to 1%. For the analysis, identified protein groups with at least one assigned unique peptide were used while removing the common contaminant and reverse hits (as defined in MaxQuant output). The label-free quantification algorithm available in MaxQuant [IV] was used for quantification of proteins identified in all three replicates in at least one group; significantly enriched proteins were selected using a t-test analysis with 5% FDR.

Ubiquitination Assays

Cell-based ubiquitination assays were performed under denaturing conditions. HEK293 cells were transfected with plasmids to overexpress the proteins as indicated in the figures. Prior to harvesting, the cells were treated with chloroquine and EGF. Cells were lysed in Ubiquitination buffer A (UBA) (8 M Urea, 0.3 M NaCl, 50 mM phosphate pH 8.0, 100 μg ml−1 NEM) to a final volume of 1 mL. Following sonication and centrifugation, lysates were incubated with Dynabeads His-tag matrices (Invitrogen) on a rotatory shaker at 4 °C overnight and processed following previous protocol [V]. The Ni^2+^–pull-down products were separated using SDS-PAGE and the immunoblots were probed for the ubiquitinated adducts using the specific antibodies. *In vitro* ubiquitination assays were performed following a previously established protocol [7]. The reaction mixtures were separated using SDS-PAGE and the ubiquitinated bands were excised, processed and analyzed by mass spectrometry to identify ubiquitinated lysine residues.

*In vitro* kinase assay

Purified active EGFR (0.1 μg) was incubated with 1 μg of GST-tagged full-length CBL variants in kinase reaction buffer (15 mM HEPES, pH 7.0, 1 mM DTT, 5 mM MgCl_2_, 5 mM MnCl_2_ and 1 mM ATP) at 30 °C for 30 min. The reactions were stopped with 2X SDS gel loading dye, separated by SDS-PAGE and subjected to immunoblotting.

RNAseq and bioinformatic analysis

RNA was extracted using the TRIzol method and the quality of extracted RNA was checked using RNA screentape on an Agilent 2200 Tapestation. An adapted method from Fisher et al [VI] was followed to prepare libraries for cluster generation and DNA sequencing using an Illumina TruSeq RNA Library Preparation Kit v2. The DNA libraries were assessed for their quantity and quality on a Qubit (Thermo Fisher Scientific) and an Agilent 2200 Tapestation (D1000 screentape), respectively. A High Output 75 cycles kit (2x36cycles, paired end reads, single index) was used to run the libraries on an Illumina Next Seq 500. Fastq version 0.10.1 (http://www.bioinformatics.babraham.ac.uk/projects/fastqc/) and fastq version 0.4.2 (http://www.bioinformatics.babraham.ac.uk/projects/fastq_screen/) were used to assess the raw RNAseq files for quality. Paired-end RNAseq reads were aligned to the mouse genome (GRCm38) with Bowtie version 2.2.4.0 [VII] and tophat2 version 2.0.13 [VIII]. Determination of expression levels and statistical analyses were done with a combination of HTSeq version 0.9.1 (https://www.huber.embl.deusers/anders/HTSeq/doc/overview.html), the R environment, version 3.1.1 within the Bioconductor data analysis suite; differential gene expression analysis was based on the negative binomial distribution using the DESeq2 [IX] package.

cDNA Synthesis and Quantitative Real-Time PCR

RNA was extracted from the samples using the TRIzol method and the quality of RNA was assessed using agarose gel electrophoresis and A260/280 measurements. Using the High capacity cDNA reverse transcription kit (Thermo Fisher Scientific), cDNA was synthesized from the high quality RNA. Quantitative PCR reactions containing 200 ng cDNA and SYBR Green master mix (Thermo Fisher Scientific) were run on an Applied Biosystems 7500 Fast Real Time PCR system. As an internal control, 18S rRNA was used in all the experiments. The collected data were subsequently plotted in PRISM.

Cdc42 activation assay

Cdc42 activation assays were performed following the manufacturer’s protocol. Briefly, lysates (500 μL at 1 mg/ml) from HEK293 cells overexpressing WT-CBL, CBL-70Z or CBL-Y371S with or without Wortmannin treatment were incubated with glutathione resin and GST-PAK1-PBD or GST alone and incubated overnight at 4 °C on a rotatory shaker. The following day, bound active Cdc42 was eluted from each combination with 2X SDS gel loading dye, separated by SDS-PAGE and immunoblotted to check for bound active Cdc42 using a Cdc42 Mouse mAb.

Recombinant protein purification

Full length GST-CIN85 was expressed and purified using glutathione-affinity chromatography, followed by anion exchange and gel filtration chromatography. GST-tagged full-length CBL, CBL-70Z and CBL-Y371S were purified using glutathione-affinity chromatography followed by anion exchange chromatography. For surface plasmon resonance (SPR) analysis, the GST-tagged ligands (GST, GST-CIN85, GST-CIN85-SH3A (1-58), GST-CIN85-SH3B (98-157), GST-CIN85-SH3C (263-328), GST-CBL-WT (47-435) and GST-CBL-Y371S (47-435) were purified using glutathione-affinity chromatography. For *in vitro* ubiquitination assay, *Arabidopsis thaliana* Uba1, UbcH5B and Ub were purified as described previously [7]. EGFR was purified from A-431 cells as described previously [7]. Protein concentrations were measured by Bio-Rad protein assay and Ub concentration was determined by absorbance at 280 nm.

SPR Binding Assays

The SPR experiments were performed with CM-5 chips (GE Healthcare) on a Biacore T200 at 25 °C. GST-tagged proteins were captured onto a CM-5 chip coupled with anti-GST to 1000-2000 response units as described previously [7]. The analytes were diluted serially in running buffer (25 mM Tris-HCl, pH 7.6, 150 mM NaCl, 1 mM DTT and 0.005% (v/v) Tween-20). Concentration ranges as indicated in Supplementary Fig. S2 were used to measure binding. Differences in signal between GST-protein and GST alone are reported. The data were analyzed by steady-state affinity analysis using Biacore T200 BIAevaluation (GE Healthcare). Representative sensorgrams were prepared in GraphPad Prism (version 8.1.0).

Breast cancer tissue microarray

Breast cancer tissue array (BC081116a; US Biomax Inc.) contained 100 breast invasive ductal carcinoma samples, 6 adjacent normal breast tissue (NAT) samples and 4 breast adenosis samples (one core per case) further categorized into 10 NAT, 7 stage-I, 77 stage-II and 16 stage-III cases (Supplementary Table 4). Following IHC, expression of CIN85, CBL and pCBL were analyzed using HALO software, representative images exported and H-scoring performed manually from fully intact cores. The actual core numbers used for quantification for CIN85, CBL and pCBL for each stage after IHC are mentioned in the figure legends. Details for patient consents and ethical standards can be obtained from US Biomax Inc.

Cell proliferation assay

MDA-MB-231 cells were seeded in 96-well plates and treated with the following peptides: 1 μM Atennapedia alone (control) and in the presence of PepC1 or PepC2 as indicated in the Fig. 6g. The cells in each well were allowed to grow for 48 hours and assayed for proliferation by incubating with equal volumes of CyQUANT direct cell proliferation (Thermo Fisher Scientific) reagent for 1 h. After 1 h, a Tecan multimode plate reader was used to measure the fluorescence with excitation at 480 nm and emission detection at 535 nm. The experiment was performed in triplicate.

Supplemental References

I Paul I, Ahmed SF, Bhowmik A, Deb S, Ghosh MK. The ubiquitin ligase CHIP regulates c-Myc stability and transcriptional activity. Oncogene. 2013;32:1284-95.

II Unbekandt M, Belshaw S, Bower J, Clarke M, Cordes J, Crighton D et al. Discovery of Potent and Selective MRCK Inhibitors with Therapeutic Effect on Skin Cancer. Cancer Res. 2018;78:2096-114.

III Hubner NC, Bird AW, Cox J, Splettstoesser B, Bandilla P, Poser I et al. Quantitative proteomics combined with BAC TransgeneOmics reveals in vivo protein interactions. J Cell Biol. 2010;189:739-54.

IV Cox J, Hein MY, Luber CA, Paron I, Nagaraj N, Mann M. Accurate proteome-wide label-free quantification by delayed normalization and maximal peptide ratio extraction, termed MaxLFQ. Mol Cell Proteomics. 2014;13:2513-26.

V Magnussen HM, Ahmed SF, Sibbet GJ, Hristova VA, Nomura K, Hock AK et al. Structural basis for DNA damage-induced phosphoregulation of MDM2 RING domain. Nat Commun. 2020;11:2094.

VI Fisher S, Barry A, Abreu J, Minie B, Nolan J, Delorey TM et al. A scalable, fully automated process for construction of sequence-ready human exome targeted capture libraries. Genome Biol. 2011;12:R1.

VII Langmead B, Salzberg SL. Fast gapped-read alignment with Bowtie 2. Nat Methods. 2012;9:357-9.

VIII Kim D, Pertea G, Trapnell C, Pimentel H, Kelley R, Salzberg SL. TopHat2: accurate alignment of transcriptomes in the presence of insertions, deletions and gene fusions. Genome Biol. 2013;14:R36.

IX Anders S, Huber W. Differential expression analysis for sequence count data.

Genome Biol. 2010;11:R106.
